# Accurate vertical ionization energy and work function determinations of liquid water and aqueous solutions†

**DOI:** 10.1039/d1sc01908b

**Published:** 2021-07-02

**Authors:** Stephan Thürmer, Sebastian Malerz, Florian Trinter, Uwe Hergenhahn, Chin Lee, Daniel M. Neumark, Gerard Meijer, Bernd Winter, Iain Wilkinson

**Affiliations:** Department of Chemistry, Graduate School of Science, Kyoto University Kitashirakawa-Oiwakecho, Sakyo-Ku Kyoto 606-8502 Japan thuermer@kuchem.kyoto-u.ac.jp; Molecular Physics Department, Fritz-Haber-Institut der Max-Planck-Gesellschaft Faradayweg 4-6 14195 Berlin Germany winter@fhi-berlin.mpg.de; Institut für Kernphysik, Goethe-Universität Max-von-Laue-Straße 1 60438 Frankfurt am Main Germany; Chemical Sciences Division, Lawrence Berkeley National Laboratory Berkeley CA 94720 USA; Department of Chemistry, University of California Berkeley CA 94720 USA; Department of Locally-Sensitive & Time-Resolved Spectroscopy, Helmholtz-Zentrum Berlin für Materialien und Energie Hahn-Meitner-Platz 1 14109 Berlin Germany iain.wilkinson@helmholtz-berlin.de

## Abstract

The absolute-scale electronic energetics of liquid water and aqueous solutions, both in the bulk and at associated interfaces, are the central determiners of water-based chemistry. However, such information is generally experimentally inaccessible. Here we demonstrate that a refined implementation of the liquid microjet photoelectron spectroscopy (PES) technique can be adopted to address this. Implementing concepts from condensed matter physics, we establish novel all-liquid-phase vacuum and equilibrated solution–metal-electrode Fermi level referencing procedures. This enables the precise and accurate determination of previously elusive water solvent and solute vertical ionization energies, VIEs. Notably, this includes quantification of solute-induced perturbations of water's electronic energetics and VIE definition on an absolute and universal chemical potential scale. Defining and applying these procedures over a broad range of ionization energies, we accurately and respectively determine the VIE and oxidative stability of liquid water as 11.33 ± 0.03 eV and 6.60 ± 0.08 eV with respect to its liquid-vacuum-interface potential and Fermi level. Combining our referencing schemes, we accurately determine the work function of liquid water as 4.73 ± 0.09 eV. Further, applying our novel approach to a pair of exemplary aqueous solutions, we extract absolute VIEs of aqueous iodide anions, reaffirm the robustness of liquid water's electronic structure to high bulk salt concentrations (2 M sodium iodide), and quantify reference-level dependent reductions of water's VIE and a 0.48 ± 0.13 eV contraction of the solution's work function upon partial hydration of a known surfactant (25 mM tetrabutylammonium iodide). Our combined experimental accomplishments mark a major advance in our ability to quantify electronic–structure interactions and chemical reactivity in liquid water, which now explicitly extends to the measurement of absolute-scale bulk and interfacial solution energetics, including those of relevance to aqueous electrochemical processes.

## Abstract 2

A generalised liquid-phase photoelectron spectroscopy approach is reported, allowing accurate, absolute energy scale ionisation energies of liquid water and aqueous solutions, as well as liquid water's work function to be reported.

## Graphical abstract

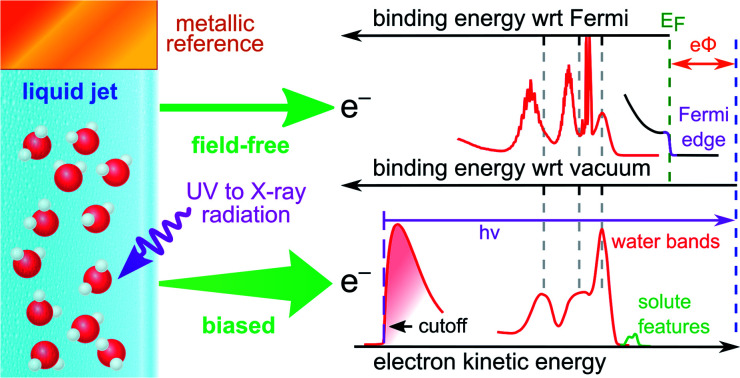

## Introduction

Knowledge of the electronic structure of liquid water is a prerequisite to understand how water molecules interact with each other and with dissolved solutes in aqueous solution. Here, the valence electrons play a key role because their energetics govern chemical reactions.^1^ One quantity of particular interest is water's lowest vertical ionization energy, VIE (or equivalently vertical binding energy, VBE), which is a measure of the propensity to detach an electron under equilibrium conditions and thus determines chemical reactivity.^2^ More precisely, VIE_vac_, where the ‘vac’ subscript refers to energetic referencing with respect to vacuum, is the most probable energy associated with vertical promotion of an electron into the vacuum, *i.e.*, without giving it any excess energy, and with no nuclear rearrangement being involved. Such VIE_vac_ values are most readily accessed using photoelectron spectroscopy (PES) – usually from gases, molecular liquids, or molecular solids – and are identified as the maximum intensities of primary, directly-produced photoelectron peaks.

Generally, in the condensed phase, PES features cannot be correlated with isolated molecular states, but are instead considered, particularly in crystalline samples, to arise from band structures, dense collections of states born from extended inter-atomic interactions.‡‡Note that the term ‘band’ for the assignment of ‘spectral bands’ in molecular spectroscopy has a different meaning to that applied within the band-structure context of condensed matter. Broad PES features are most often observed, from which it is often impossible to reliably extract valence VIE values. However, in molecular liquids and molecular solids, peak structures usually remain isolable, with associated VIE_vac_ values regularly being extracted and described within a molecular physics framework. Here, simple molecular orbital formalisms are adopted, with the peak structures ascribed to the liberation of electrons from specific orbitals. Adopting such an approach, the molecular orbitals of the water monomer have been considered to be only weakly perturbed by hydrogen bonding in the liquid phase, without specific regard for inter-monomer interactions or explicit consideration of the aqueous interface. The lowest VIE_vac_ value of water has correspondingly been assigned to ionization of the non-bonding 1b_1_ highest occupied molecular orbital (HOMO) in the gas,^3^ liquid,^4^ and solid^5^ phases. In fact, this molecular electronic structure description, and a vacuum level energy referencing approach, has almost exclusively been adopted in the interpretation of liquid-phase PES spectra.^2,6,7^ This is in spite of liquid water (and aqueous solutions) exhibiting both molecular^4,8–11^ and dispersed ‘band’^7,8,12–17^ electronic structure signatures. Naturally, this raises the questions of how liquid water should be placed between the aforementioned molecular and condensed matter conceptual frameworks, and specifically what can be learned by applying concepts from the latter to the PES of liquid water and aqueous solutions.

Within a condensed-matter framework and at thermodynamic equilibrium, the available states (or bands) of a system, are separated into occupied and unoccupied components around the Fermi level, *E*_F_. As a precisely defined thermodynamic quantity, energy referencing with respect to *E*_F_ engenders direct comparison of system energetics between condensed-phase samples and the ready relation of those energetics to additional thermodynamic quantities. Such a useful energetic reference is readily accessible in metals using PES, where *E*_F_ lies within the available states and defines the upper electronic occupation level. In contrast, in semi-conductors, *E*_F_ is placed within a ‘forbidden’ band gap (devoid of states) and is thus, directly at least, inaccessible using the PES technique; *E*_F_ is notably not an electronic state that can donate or accept electrons here, rather it corresponds to a thermodynamic energy level. Liquid water, like most other liquids, can be classified as a wide-band-gap semiconductor,^18–20^ with a generally inaccessible Fermi level. Upon first consideration, liquid water may, therefore, seem unsuited to an *E*_F_ energy referencing scheme. Clearly, the solid-state custom of indirectly energy-referencing semi-conductor PES spectra to *E*_F_*via* a metallic reference sample is much more difficult to apply to volatile and potentially charged aqueous-phase samples.

The VIE_vac_ values predominantly considered in liquid-phase PES experiments so far, as well as any VIE values determined with respect to *E*_F_, VIE_EF_, arise from the cumulative energetics of a photoemission process. This includes the effects of collective phenomena (hydrogen bonding, inhomogeneous broadening *etc.*), electron transport, and an interface (typically liquid-vacuum),^21–23^ where the latter has yet to be explicitly addressed in liquid-phase PES studies. In liquid water, the ionization energies are specifically affected by inhomogeneous and fluxional intermolecular hydrogen bonding interactions. Here, the associated energetics vary over the transition region spanning the aqueous bulk and the liquid interface through which photoelectrons must traverse to escape into vacuum. These properties are closely related to distinctive condensed-matter system descriptors that are of particular relevance to photoemission, such as electrical conductivity, chemical potential (*μ*, equivalent to *E*_F_), electrochemical potential (*μ̄*), work function (e*Φ*), surface dipole, and surface (dipole) potential (*χ*^d^ or e*φ*_outer_).^24–26^ We present an overview of the relations between these parameters, with a focus on the liquid water system, in Fig. SI-1 of the ESI† and note that even after many years of aqueous-phase PES research, previous evaluations of liquid water's (lowest) VIE_vac_ values^4,27–29^ have barely considered these condensed matter descriptors. In other words, more differential probes of the bulk and interfacial electronic structure properties of liquid water and aqueous solutions have barely been addressed in PES experiments.§§Although the importance of the determination of the cutoff energy in liquid-jet photoelectron spectra, with the aim of quantifying work functions from aqueous solutions, has been accented already in 2003,^94^ this approach was barely further considered for the subsequent 10–15 years. Arguably, the reason is a combination of the gas-phase-references being such an easy and convenient (although problematic) method, and difficulties in the technical realization of low-energy electron detection with liquid-jet PES setups. For a significant time, and this remains true in many cases, LJ-PES (liquid jet photoelectron spectroscopy) experiments with HEAs were barely designed to detect low-kinetic energy electrons, typically due to insufficient magnetic shielding and likely because it had yet to be demonstrated that liquid phase PES is capable of accessing characteristic condensed-matter properties. Finally, the ability to properly apply a bias voltage to a liquid jet had to be thoroughly explored, an issue with remaining open questions, such as the degree of the deleterious effects of biasing an entire sample delivery assembly, as opposed to just the liquid stream.

We show here that the application of concepts from condensed-matter physics to liquid-jet (LJ) PES enables a significant expansion of our understanding of the electronic structure of liquid water. Towards that wider goal we pronounce two immediate aims. The first is to determine an accurate value of the lowest vacuum-level-referenced VIE of liquid water, VIE_vac,1b1(l)_ (equivalent to its HOMO or 1b_1_ orbital ionization energy). Perhaps surprisingly, after more than 15 years of research, the value of this quantity remains controversial, mirroring key shortcomings in previous experiments. We address these deficiencies here and identify the need for additional spectroscopic information. For this particular task, the missing quantity is the (yet-to-be-discussed, although previously alluded to^4,30,31^) low-energy electron cutoff in the liquid-water PES spectrum, a commonly measured parameter in solid-state PES.^23,32–35^ Motivated by a possible depth dependence of VIE_vac,1b1(l)_ (*i.e.*, of neat water), we utilize the cutoff spectral feature to report the first systematic study of VIE_vac,1b1(l)_ over a large range of photon energies, spanning the (vacuum) ionization threshold region up to more than 900 eV above it. We apply the same concepts to determine water's lowest VIE from exemplary aqueous solutions, VIE_vac,1b1(sol)_, in addition, *i.e.*, detecting the solute-induced effect on water's electronic structure. We similarly demonstrate how to extract the VIEs of aqueous solutes, VIE_vac,solute_, over a broad range of concentrations. Our second principal objective is to demonstrate how to measure *E*_F_ and e*Φ* of liquid water and aqueous solutions. We will discuss the meaning and importance of *E*_F_ in the case of the liquid water system, with the main goal of obtaining liquid-phase VIEs referenced to its Fermi level (VIE_EF_), including those of neat water (VIE_EF,1b1_), the aqueous solvent (VIE_EF,1b1(sol)_), and associated solutes (VIE_EF,solute_). The successful implementation of this alternative aqueous-phase PES energy referencing scheme permits a direct comparison between liquid- and solid-phase PES results. It further enables more direct derivation of additional thermodynamic quantities from aqueous-phase VIE measurements, including redox energetics. The combination of the VIE_EF_ information with respective VIE_vac_ measurement results allows e*Φ* values to be derived and the explicit characterization and quantification of aqueous interfacial effects. Finally, we evaluate the challenges in characterizing Fermi level alignment between solutions and reference metals based on the currently available experimental methods, as we start to bridge the gap between aqueous-phase and solid-phase PES.

## LJ-PES from water and aqueous solution

### The common experimental approach

**Fig. 1:**
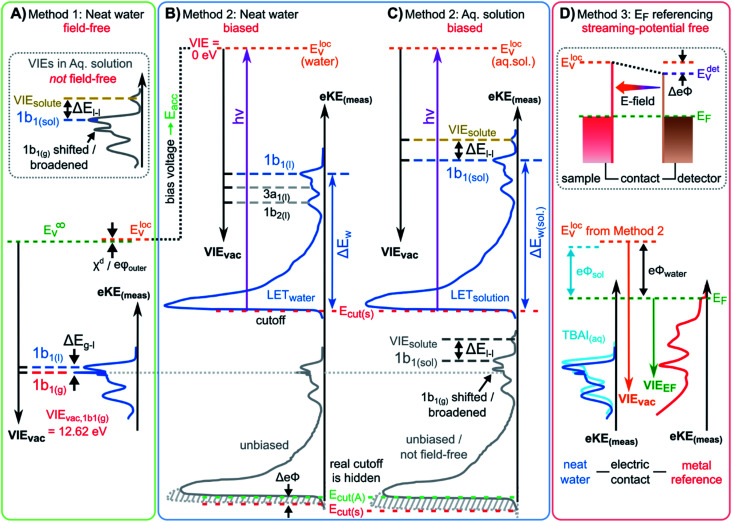
Schematic electronic energetics for each experimental method described in the main body of the text. (A) Both gas- and liquid-phase water spectral features are measured together on the eKE_(meas)_ scale under field-free conditions (blue spectrum), which makes it possible to use the known gas-phase VIE values (red) as an energy reference; ionization energies, VIE_vac_, are referenced to the vacuum level at infinity, *E*_v_^*∞*^. The inset shows the commonly adopted extension of Method 1 to reference solute VIE values by determining the solute peak's energetic distance to the liquid water 1b_1_ peak, Δ*E*_l-l_, and (generally inappropriately) using the VIE_vac,1b1_ value of neat water as a reference value. Any possible changes of VIE_vac,1b1_ in a solution or the aqueous e*Φ* are disregarded in this approach. (B) A bias applied to the LJ shifts all liquid features under the influence of an accelerating field, *E*_acc_ (blue spectrum); the gas-phase PE signal is smeared out and does not appear here. Biasing reveals the full LET curve and cutoff energy of the sample spectrum, *E*_cut(s)_. Without bias (grey spectrum), the real cutoff is obscured by the work-function difference between the liquid and analyzer, Δe*Φ*, and one would instead measure a setup-dependent cutoff energy, *E*_cut(A)_. *E*_cut(s)_ constitutes a low-energy limit for photoelectrons to still overcome the liquid-surface barrier, and is thus connected to the local vacuum level above the LJ surface, *E*^loc^_v_. The precisely known photon energy *hν* (vertical purple arrow) is used to map *E*^loc^_v_ onto the measured spectrum and define the VIE_vac_ scale. Note that in general *E*^loc^_v_ will deviate from *E*_v_^*∞*^ due to the intrinsic surface potential *χ*^d^/e*φ*_outer_ (see panel A and the text for details). Any extrinsic potentials are irrelevant in the applied bias case because the only relevant quantity is the energetic separation of the PE features from *E*_cut_, Δ*E*_w_ (blue arrow). (C) As for (B) but for an arbitrary aqueous solution; here, the spectra are arbitrarily aligned to the cutoff, which at the same time aligns *E*^loc^_v_. Changes in Δ*E*_w_ directly translate to changes in the VIE. The lower part of this panel shows the full unbiased spectrum (compare to the spectra shown in the inset in panel A and bottom part of panel B). (D) The liquid water spectrum (dark blue) is energy-referenced to a common Fermi level, *E*_F_, which defines the ionization energy scale with respect to Fermi, VIE_EF_. This is achieved by separately measuring a metallic sample (red spectrum) in electrical contact and equilibrium with the liquid. The liquid-phase measurements must be performed with a sufficient amount of dissolved electrolyte to suppress the streaming potential and assure good conductivity. The Fermi-alignment with the apparatus leads to an offset of the local vacuum potentials as shown in the top inset in panel D. This creates an intrinsic potential difference due to the generally different e*Φ* values between the sample and the apparatus (detector). Thus, the measurement is usually not performed under field-free conditions, unlike Method 1. The difference between the VIE_vac_ and VIE_EF_ scales yields water's work function, e*Φ*_water_. We additionally sketch (light blue), the situation where e*Φ* changes and the valence spectrum shifts with respect to *E*_F_ upon build-up of a surface dipole arising from adsorbed interfacial anions and cations (here, representative of a surface-active TBAI aqueous solution; although this latter detail is not depicted). TBAI_(aq)_ is known to exhibit a pronounced surface-dipole layer comprised of slightly spatially separated maxima in the TBA^+^ and I^−^ concentration profiles,^86^ which may lead to a reduction in e*Φ*. This in turn would shift the position of *E*^loc^_v_ of the TBAI solution with respect to the metallic sample.

We begin with short overviews of the LJ-PES technique, the commonly adopted LJ-PES vacuum energy referencing method, and the current challenges in measuring accurate VIE_vac_ values of liquid water and solutions more generally. We also present some useful considerations on the application of a VIE scale to condensed-phase PE spectra in ESI Section 1,† which we apply from here onwards. Since the experimental breakthrough in detecting photoelectron spectra from aqueous solutions, marked by the availability of vacuum liquid microjets^36,37^ over 20 years ago, a flurry of LJ-PES measurements has been conducted. Such measurements have greatly advanced our understanding of the electronic structure of aqueous solutions, in the bulk and at the solution–vacuum interface, as has recently been reviewed.^38^ Notably, however, aside from very few exceptions, previous LJ-PES measurements have garnered the bare minimum spectral information, for which it has sufficed to detect a narrow range of electron kinetic energies, eKEs, of the emitted photoelectron distributions. For example, from aqueous LJs and their evaporating vapor layer, the characteristic eKEs of a solute or liquid water ionization feature of interest, VIE_vac,(l)_, and the lowest energy gas-phase ionization peak, VIE_vac,1b1(g)_, can be simultaneously determined. The latter value is accurately known (12.621 ± 0.008 eV),^3^ and from the difference of the measured peak positions, Δ*E*_g-l_ = VIE_vac,1b1(g)_ – VIE_vac,1b1(l)_, VIE_vac,1b1(l)_ can (in principle) be determined.^4,28,36^ Adopting this procedure, here referred to as Method 1, vacuum-level energy referencing and production of the aqueous-phase photoemission spectrum is achieved without the need for further information. This simple and highly convenient molecular-physics approach, which is however challenging to accurately apply, as we will show below, is illustrated in Fig. 1A. There, we depict the measured valence photoemission spectrum of liquid water, *i.e.*, the kinetic energy distribution curve of the emitted photoelectrons, and the energy difference, Δ*E*_g-l_, between the lowest energy liquid-, 1b_1(l)_, and gas-phase, 1b_1(g)_, water ionization features.

LJ-PES experiments commonly use rather high photon energies, typically some tens or more electron volts above the relevant ionization thresholds. Such photon energies sufficiently separate directly-produced photoelectron peaks from the low-energy background of inelastically scattered electrons^23^ and minimize scattering-induced distortions of the PE peaks themselves^30^ (owing to the fact that electron scattering is almost exclusively governed by electronic excitations at such photon and kinetic energies^39^). The vast majority of LJ-PES studies have adopted such photon energies to establish solute *core*-level energies, with the measured chemical shifts serving as a reporter of changes in the chemical environment. Small discrepancies in absolute core-level energies among different laboratories typically have little consequence on the main observations and derived statements. Similarly, the large body of studies of Auger decay and other autoionization processes from the aqueous phase^40–43^ would be barely affected by small uncertainties in absolute electron energies. This is in contrast to the situation with valence LJ-PES, which has been far less explored^2,44,45^ despite the primary importance of the lowest-ionization energies in driving aqueous-phase chemistry.^2^ In this case, after more than 15 years of active high-energy-resolution LJ-PES research,^38,43^ and with concomitant advancement of aqueous electronic structure calculations and spectral simulation methods,^8,9,41,46–52^ an experimental advance and alternative terminology must be adopted to enable unequivocal and accurate valence VIE determinations with respect to the vacuum level. Related developments are needed to permit *E*_F_ (or system chemical potential) energy referencing of LJ-PES spectra, robust e*Φ* extractions from liquid samples, and direct comparisons of liquid- and solid-phase absolute-scale electronic energetics.

To understand the shortcoming of previous studies it is sufficient to discuss why the exact value of VIE_vac,1b1(l)_ from neat water continues to be debated, spanning a 0.5 eV range between 11.16 ± 0.04 eV ^4^ and 11.67 ± 0.15 eV.^29^ All previously reported reference values were obtained using Method 1, from a mere Δ*E*_g-l_ measurement which neither requires the determination of absolute eKEs nor an exact calibration of the applied photon energy. However, a seemingly simple measurement of Δ*E*_g-l_ is difficult to accomplish due to the multiple sample charging effects and contact-potential differences that occur in LJ spectrometer systems (see the Discussion in Section 2 in the ESI and ref. 7, 28, 53 and 54). Accurate Δ*E*_g-l_ measurements are further complicated by the temporal variation of surface potentials within LJ-PES apparatuses, due to the continuous evaporation of LJs and the establishment of stable, adsorbed surface layers within spectrometers. All of these perturbing influences generate electric fields between the sample and the electron detector, which affect the photoelectrons from the gas and liquid phases differently and have to be precisely accounted for to record the ‘true’ (*i.e.*, undisturbed) Δ*E*_g-l_ value. As knowledge about the relevant effects and methods for their elimination continues to evolve,^28,53,54^ reported Δ*E*_g-l_ values, and thus deduced VIE_vac,1b1(l)_ values continue to vary from laboratory to laboratory, which explains the scatter of the reported energies mentioned above.

Efforts to measure accurate Δ*E*_g-l_ values center around the minimization or even elimination of the effects of perturbing potentials, compensating electrokinetic and other forms of charging of the LJ and other local potentials to achieve what we refer to as ‘field-free’ conditions. The primarily adopted method achieves this by implementing a small but precisely determined salt concentration in water at a given solution flow rate and temperature.^28^ Alternatively, the provision of field-free conditions through application of a compensating bias voltage to a LJ has been discussed.^29,31^ In spite of such compensation efforts, the stabilization of spectrometer potentials occurs on the order of tens of minutes to hours after LJs are started or experimental parameters are adjusted, for example, when cold trap coolant is replenished. As we show in Fig. SI-2,† the apparatus potentials change dramatically (more than 100 meV) over time upon introducing water vapor into the experimental vacuum chamber, while eventually settling into an equilibrium. Unsurprisingly, these effects are difficult to quantify for a given experimental setup and operational conditions.

Here we highlight another potentially crucial and barely realized issue with Method 1, namely the meaning of the vacuum level. We have introduced VIE_vac_ above without providing a sufficiently accurate definition of the relevant vacuum level in a LJ-PES experiment. VIE_vac,1b1(g)_ (like any other gas-phase ionization energy) is necessarily referenced to the vacuum level at infinity, *E*_v_^*∞*^ (used in Fig. 1A), and corresponds to the potential energy of the photoelectron at rest and at infinite separation from the photoionized sample.^24^ In all previous LJ experiments, it has been implied that this same vacuum level is applicable and accessible upon ionization of liquid water, with existing VIE_vac,1b1(l)_ values being consequently referenced to *E*_v_^*∞*^*via* VIE_vac,1b1(g)_. Adopting this assumption, the most probable (vertical) gas- and liquid-phase ionization energies have been taken as the maxima of the gas- and liquid-phase photoelectron (PE) peak fits within an encompassing spectrum. The consequences of this assumption will be further discussed below.

A yet further encountered and momentous oversight in previous LJ-PES studies is the determination of aqueous-phase solute VIE_vac_ values (VIE_vac,solute_) with reference to predetermined VIE_vac,1b1(l)_ values measured from neat water, ideally under field-free conditions. That is, in (almost) all previous LJ-PES valence and a number of core-level studies spanning a broad range of aqueous solutions,^2,38^ the VIE_vac,1b1(l)_ value (*i.e.*, from neat water) has in fact been used (as is) to calibrate VIE_vac,solute_ values. Specifically, the energy difference between the solute PE peak position and lowest-energy solvent PE peak position, VIE_vac,1b1(sol)_, has been used, under the generally erroneous assumption that VIE_vac,1b1(sol)_ = VIE_vac,1b1(l)_. This is illustrated in the inset of Fig. 1A, where Δ*E*_l-l_ is the measured energy difference between two liquid-phase peaks, the lowest ionization energy, 1b_1(l)_, solvent peak and a solute peak. This energy referencing is generally rendered meaningless when non-negligible solvent–solute interactions and/or solute-induced interfacial electronic structure changes occur. In core-level studies, often the O 1s core-level energy (established for neat water only, again under field-free conditions)^55^ has alternatively been used to similarly energy-reference VIE_vac,solute_ values, with the same fundamental deficiencies. Such practices imply that solute-induced water electronic structure and solution e*Φ* changes do not occur, an assumption which has no rigorous foundation and may easily lead to quantitative failure of this extended implementation of Method 1, as recently discussed in ref. 7 and enunciated in ref. 31.

Alternatively, but equally problematic, one could strive for the determination of VIE_vac,solute_ with reference to VIE_vac,1b1(g)_, using the basic variant of Method 1, *i.e.*, the hypothetical field-free variant of what is shown in the main section of Fig. 1A. Yet, as detailed above, only if the region between the LJ interface and detector were field-free, could the measured electron energies from the gas-phase molecules be directly related to those from the liquid phase. For almost all solutions, field-free conditions are not or cannot be established in the experiment, and the same problems remain as for neat water. Thus, any additional field introduced to the solution – *via* electrokinetic charging, ionization-induced charging, or surface dipoles – renders the direct Δ*E*_g-l_ energy referencing for the solute peaks *via* (extrinsically field-free) values of VIE_vac_, 1b_1(g)_ questionable. With Method 1, the relative contributions to the sample charging cannot be quantified, and field-free conditions thus only arise from the fortunate mutual compensation of any charging and/or differential e*Φ* effects.

Furthermore, and more fundamentally, the effects of any intrinsic and non-negligible interfacial dipole potential, *χ*^d^, at the water liquid–vapor-phase interface^56^ could lead to intrinsic offsets of Δ*E*_g-l_ from its true value, potentially compromising energy referencing Method 1. The value of the liquid water interfacial surface dipole potential has yet to be directly experimentally determined, although it has been inferred to amount to a few tens of meV in neat water,^57,58^ with associated theoretical predictions^56,59–62^ of *χ*^d^ varying significantly. In aqueous solutions, the value of *χ*^d^ is expected to be highly solute- and concentration-dependent,^56^ calling the extended Method 1 energy referencing schemes for aqueous solutions further into question. Hence, to uniquely and generally interrogate both solute and solvent electronic structure on an absolute energy scale, a novel and robust experimental procedure that relies on an energy reference other than VIE_vac,1b1(g)_ must be developed.

### Condensed-matter approach and absolute energy reference

Above we have seen that an approximate value of VIE_vac,1b1(l)_ from neat liquid water – with up to 0.5 eV uncertainty, depending on the care taken to compensate extrinsic potentials – can be obtained with the conceptually simple Method 1 (Fig. 1A). Adopting a more robust, absolute energy referencing method afforded using the low-energy photoelectron signal cutoff, *E*_cut_, as widely applied in solid-state PES,^23,32–35^ the field-free requirement for accurate VIE_vac_ measurements is lifted. We now consider the associated energy-level diagram shown in Fig. 1B to illustrate this more robust and generally applicable experimental approach. In fact, as a requirement for an accurate VIE_vac,1b1(l)_ (or alternative liquid-phase VIE_vac_) determination, a negative bias voltage should be deliberately applied between the LJ and the electron analyzer orifice, imparting a well-defined additional eKE to the liquid-phase photoelectrons *via* an accelerating field, *E*_acc_ (indicated as black dotted line in Fig. 1B); we explain why the application of a bias voltage is indispensable below. Hence, a prerequisite for this approach is a sufficiently electrically conductive sample that supports the applied bias, held in direct electrical contact with the electron analyzer *via* a stable DC power supply. Not only does this allow the unequivocal resolution of the true value of VIE_vac,1b1(l)_ from neat water, the respective value (as well as any associated solute VIE_vac,solute_) can also be accessed from *any* aqueous solution. In fact, the same methodology is also directly applicable more generally, for example, to organic solutions. Moreover, novel information on the solution–vacuum interface is conveniently provided.

The full LJ-PES spectrum from neat liquid water is sketched in Fig. 1B. The case of a photon energy sufficiently in excess of the first three ionizing transition thresholds of liquid water (1b_1_^−1^, 3a_1_^−1^, and 1b_2_^−1^ in a molecular-physics description) to yield undistorted primary photoelectron peaks is illustrated. Spectra associated with grounded (grey curve) and negatively biased (blue curve) liquid samples are shown. In the biased case, the entire liquid-phase spectrum experiences a rigid energy shift, equivalent to the negative bias voltage (see Fig. SI-3† for an experimental example of this effect). The exact value of the bias voltage is rather irrelevant for the present purpose. Unlike in Fig. 1A, the spectra in Fig. 1B encompass the full low-KE tail, LET, which terminates the spectrum at eKE = 0 eV.¶¶We will refer to the inelastic scattering tail as the low-KE tail or LET curve throughout the manuscript, in contrast to the often-used term secondary electron energy distribution (SEED) curve described in previous studies. This is consistent with the fact that at lower photon energies, when the kinetic energy of the primary electron is too low for efficient secondary electron generation *via*, *e.g*., impact ionization, the inelastic scattering background is not fully comprised of secondary electrons. Thus, the term SEED cannot be used for aqueous solution spectra recorded at ∼20 eV photon energies and below.^30^ The term LET is adopted to avoid misleading connotations about the origin of this low energy signal. The LET comprises electrons which have lost most of their energy due to various inelastic scattering processes, and have just enough energy to overcome the surface barrier of the sample. They are accordingly expelled with quasi-zero kinetic energy, signified here by the *E*_cut_ label, with *E*_cut_ defining the energetic zero from the perspective of a photoelectron leaving the sample.^35^ Hence, the concurrent measurement of *E*_cut_ (=0 eV) and the VIE_vac_ values of interest – such as VIE_vac,1b1(l)_ – allows the unique and self-consistent assignment of an eKE reference to the LJ-PES data, irrespective of *any* perturbing potentials, intrinsic or extrinsic. From Fig. 1B, it is seen that the eKE of the 1b_1(l)_ peak can be accurately determined *via* its energy separation from *E*_cut_, *i.e.*, the spectral width, Δ*E*_w_. The associated VIE is correspondingly determined as VIE_vac,1b1(l)_ = *hν* − KE_1b1(l),_ where *E*_cut_ is set to 0 eV and it is implied that the photon energy is precisely known (we discuss procedures to precisely determine *hν* for various light sources in the ESI Section 3). This procedure of measuring the *full* PES spectrum (or, at least, the LET region and the PE features of interest under the same conditions) will be referred to as Method 2 in the following. Importantly, gas-phase peaks or referral to VIE_vac,1b1(g)_ are now irrelevant for the accurate extraction of VIE_vac,1b1(l)_, or any other solvent or solute VIE. Furthermore, a favorable side effect of applying a high enough bias voltage is that the liquid-phase PE spectrum can be obtained essentially free from otherwise overlapping gas-phase signal, as is indicated by the missing sharp 1b_1(g)_ peak in the blue curve in Fig. 1B. In that case, the varying electrostatic potential between the biased liquid sample and the grounded electron analyzer results in a gas-phase peak broadening and a differential gas–liquid shift which is sufficient to move the gas-phase peak centers out of the liquid phase spectrum. Thus, the gas-phase features can almost be fully pushed out of the spectral range of interest. Notably, however, it is impossible to fully suppress the gas-phase signal at the energy position of the liquid spectrum by applying a bias, as some gas-phase molecules will always reside directly above the surface and experience the full bias potential.

We have not yet thoroughly motivated the rationale for conducting experiments on a negatively biased sample, which so far was rarely practiced in liquid-phase PES. In the case of an unbiased LJ, the spectrum of the LET is typically obscured by the measurement process, as the PE distribution is modified by additional scattering inside the electron analyzer and then generally arbitrarily terminated at a low-energy cutoff, *E*_cut(A)_, by the analyzer's own internal work function.^34,35^ This makes an accurate distinction of the true sample cutoff, *E*_cut(s)_, impossible. The overlapping cutoffs for the unbiased liquid-water jet are correspondingly depicted in the bottom part and grey spectrum in Fig. 1B, with this spectrum being energetically-aligned with that shown in Fig. 1A. As partially highlighted in Fig. 1B, only by applying a sufficiently large negative bias voltage to the liquid jet can the LET curve of the sample and the secondary electron signals produced in the analyzer be well separated, the arbitrary *E*_cut(A)_ threshold be far exceeded, and *E*_cut(s)_ be precisely determined.

Thus far we did not comment on the appropriate vacuum reference level for Method 2. As alluded to above, gas-phase and condensed-phase PES measurements in principle refer to different vacuum levels. This is connected to the presence of a surface, through which the photoelectrons have to traverse as the final step in a condensed-phase PE process.^23^*E*_cut_ marks the minimum energy for a photoelectron to surmount the surface barrier and be placed at rest at a point in free space just outside the surface, overcoming e*Φ* (*i.e.*, where the electron image potential at the surface drops to zero and at a distance from the surface that is much smaller than the dimensions of the surface itself).^24^ This connects all energies inferred with Method 2 to the *local* vacuum level, *E*^loc^_v_, but not necessarily to *E*_v_^*∞*^. In aqueous solutions, the offset of *E*^loc^_v_ with respect to *E*_v_^*∞*^ can be related to the outer (Volta) potential e*φ*_outer_ or *χ*^d^,^59^ note the small *E*^loc^_v_*versus E*_v_^*∞*^ difference labeled *χ*^d^/e*φ*_outer_ in Fig. 1A and B, where the panels connect. Generally, an intrinsic millivolt to volt scale dipolar surface potential, *χ*^d^, is expected to occur at the aqueous liquid–gas interface as the molecular density and hydrogen bond structure of bulk liquid water or an aqueous solution evolves from fully hydrated to partially hydrated and to increasingly isolated molecules in the gas phase. A range of experimental^57,58,63^ and theoretical^56,59–62^ studies have been performed to infer or calculate the net dipolar alignment and associated interfacial potential difference in the neat (or nearly neat) water case. While few tens of meV values have been inferred experimentally,^57,58^ a consensus on the value of *χ*^d^ at the water liquid-vapor-phase interface has yet to be reached from a theoretical perspective, and direct experimental measurements have not, to our knowledge, been reported. Relating this to the present discussion, *χ*^d^ clearly only emerges within a condensed-matter description of the aqueous-phase electronic structure. Furthermore, any non-negligible *χ*^d^ value would differentially affect electrons born at different points across the aqueous bulk to gas-phase transition region. Correspondingly, energy referencing Method 2 and the thus far adopted direct Δ*E*_g-l_ energy referencing approach, Method 1, can be expected to yield inherently different VIE_vac,1b1(l)_ values if a significant liquid water *χ*^d^ pertains.

Moving beyond our primary consideration of neat liquid water, Method 2 can also be applied without amendment to aqueous (or other) solutions, as shown in Fig. 1C. We can thus determine VIE_vac,1b1(sol)_ with the same high accuracy as VIE_vac,1b1(l)_ for neat liquid water, with the additional possibility of precisely determining other aqueous-phase solvent and solute VIEs. VIE_vac,1b1(l)_, VIE_vac,1b1(sol)_, and VIE_vac,solute_ are again obtained as VIE_(l)_ = *hν* − KE with *E*_cut_ defining zero KE. A solute-induced change of the former is seen to directly correspond to a change in the measured 1b_1_ ionization feature KE, corresponding to the different values of Δ*E*_w_ and Δ*E*_w(sol)_. We show an additional high-KE peak in Fig. 1C to exemplify the photoionization of a solute component. We emphasize that in the presence of a solute, surface potentials (in addition to the aforementioned extrinsic fields) are likely to be modified, generally making it impossible to establish the field-free conditions needed to directly apply Method 1. Its extended variant – measurement of Δ*E*_l-l_ and energy referencing to the field-free value of VIE_vac,1b1(l)_ – as has so far been utilized to obtain reference energies for VIE_vac,solute_ values, is similarly invalidated. Method 2, on the other hand, is not affected and thus permits direct access to absolute VIE changes between aqueous (or alternative) solutions for the first time. We further stress that Method 2 probes VIEs with respect to the local vacuum level *E*^loc^_v_ and that the energetic position of *E*^loc^_v_ with respect to *E*_v_^*∞*^ generally varies depending on the solution (note that the schematic biased spectra in Fig. 1C are arbitrarily aligned to the low-energy cutoff, which simultaneously aligns *E*^loc^_v_). Analogous to Fig. 1B, we illustrate the spectrum measured from an unbiased aqueous solution at the bottom of Fig. 1C, which highlights the overlapping sample and spectrometer LET curves and depicts the general inaccuracy of unbiased Δ*E*_l-l_ measurements when energy referenced using previously determined field-free, neat water VIE_vac,1b1(l)_ values, as shown in the inset of Fig. 1A.

### Fermi-level referencing and solution work functions

In the following we consider additional steps beyond the absolute, vacuum level energy referencing ability of Method 2 (Fig. 1B and C) and address the interfacial electronic structure information that becomes accessible using a condensed-matter framework and associated experimental approach. This leads us to attempt to determine *E*_F_ and e*Φ* in both water and aqueous solutions, with the latter providing a means to differentiate between solute-induced changes of (bulk or surface) liquid electronic structure or interfacial effects. Correspondingly, we briefly explain the concepts of *E*_F_ and e*Φ*. *E*_F_ is formally equivalent to the chemical potential, *μ*, and at thermodynamic equilibrium is the energy at which a (potentially hypothetical) electronic state has 50% probability of being occupied at fixed temperature and any given time. The position of *E*_F_ throughout matter in electrical equilibrium assumes the same thermodynamic value. This makes *E*_F_ an advantageous energy reference in condensed-matter spectroscopies, especially for metallic samples, in which electrons occupy states up to *E*_F_, and which can be directly measured using photoemission. *E*_F_ is conceptually connected to two additional important quantities, the electrochemical potential, *μ̄*, and the work function, e*Φ*. *μ̄* is the energy required to bring an electron at rest *at* infinity into the bulk of the material. Hence, the sum of *E*_F_ and *μ̄* is equivalent to *E*_v_^*∞*^ (and in a metal, the energy of *μ̄* with respect to *E*_F_ is equivalent to the electron affinity). In contrast, e*Φ* is the minimum energy required to remove an electron at *E*_F_, deep inside the material, and place it at rest at a point in free space just outside the surface, thus connecting to the local vacuum level, *E*^loc^_v_. *E*^loc^_v_ and e*Φ* are correspondingly local properties of a surface which can change widely depending on the surface conditions.||||In fact, it is possible to deliberately modify e*Φ*, *e.g*., by adsorption of molecules on the sample surface, which typically induces or alters a pre-existing surface dipole, the associated value of *χ*^d^, and necessarily the value of *E*^loc^_v_.^24,35,91^ This creates or modifies the energetic barrier for the photoelectrons escaping from the sample into vacuum, and can be detected as a change in KE of the emitted electrons. For a sample with a truly uncharged, amorphous, apolar surface, we note that *E*_v_^*∞*^ = *E*_F_ + *μ̄* = *E*_F_ + e*Φ*. However, if an intrinsic dipolar surface potential exists, the first equality holds, and the second generally will not. In such a case, and depending on the geometry of the liquid surface and its overlap with an ionizing light source, condensed phase VIEs or binding energies will be offset by an experimental-geometry-averaged amount with respect to *E*_v_^*∞*^ due to the average offset of *E*^loc^_v_.

Fig. 1D depicts the energetic alignment of *E*_F_ for grounded liquid water and a grounded metal, which implies electrical contact between the liquid, the metal sample, and the analyzer. The exact meaning of ‘aligning the Fermi level’ of a solid and a liquid will be detailed in the Discussion section.† To generate accurate PES results, sufficient electrical conductivity must be engineered between all of these elements while suppressing parasitic extrinsic potentials, such as the aforementioned LJ streaming potential. Under these conditions, *E*_F_ can be directly measured from a metal, as indicated by the red archetypal spectrum on the right of Fig. 1D. The water sample, which is in direct electrical contact with the metallic reference sample and the analyzer, is then separately probed under the same conditions to produce the blue water spectrum on the left of Fig. 1D (identical to that shown in Fig. 1A). Sequential PES measurements from these two samples accordingly provides a means to formally assign *E*_F_ to liquid water (as implied in Fig. 1D), and hence define the energy scale needed to determine water's ionization energy with respect to the Fermi level, VIE_EF,1b1(l)_.**Here we contrast the electronic structure of liquid water with that of metallic, ionic, or covalent macroscopic solids, where delocalized electronic states are formed *via* atomic valence energy level interactions that generate quasi-continua of energy levels, termed bands. An important consequence of this behavior is that PES spectra recorded from non-molecular systems generally exhibit broad valence features that elude association with specific VIEs. Thus, VIE is a quantity less typically encountered in a condensed-matter electronic structure context, with band edge electronic structure descriptors more commonly being reported. It is of great interest to explore how these different descriptors and experimental observables interconnect within a unified ‘band structure’ description of typical solids, liquid water, and aqueous solutions, although this is beyond the scope of the present study. Such pairwise measurements will be reported here, where extensive efforts have been made to measure the LJ sample and metal reference spectra under as similar conditions as possible, for example by recording the latter in the presence of the LJ in operation to capture any potentially distorting influences of the LJ. The measured *E*_F_ position from the metal reference sample was found to remain constant within ∼2 meV, regardless of conditions inside the vacuum chamber or whether the LJ was on or off. Despite this, our associated experimental approach, referred to in the following as Method 3, does however have a notable deficiency. As the electrons emitted from the metal are measured without crossing the solution–vacuum interface, any parasitic potentials and surface effects uniquely present on the LJ are not captured by Method 3. Extrinsic potentials, such as the streaming potential and light-induced surface charging, which are dependent on the solution and various experimental parameters, pose a new and unique challenge to the Fermi-referencing approach.††††The problem of ionization-induced charging is well-known in solid insulator studies and is usually sufficiently counteracted using neutralization instrumentation such as electron flood guns.^95^ Notably, the charging of the surface of a volatile, flowing aqueous solution in a low-vacuum environment cannot be compensated in this way. In order to accurately and generally perform the *E*_F_ referencing procedure, the electrons from the metal sample would also need to be detected following traversal of the solution–vacuum interface, for example using a PES-compatible solution-on-metal sample system incorporating a continuous solution flow (to avoid sample contamination and cumulative photo-induced degradation). With presently available experimental techniques (including electron-permeable flow cell windows^64^), such a measurement remains elusive^65^ due to the small electron mean free path in water.^66,67^ This constitutes one of the major challenges in applying PES to study water–solid interfaces. However, although an ideal *E*_F_ alignment and single-experiment *E*_F_-referenced liquid-phase PES measurement (as suggested in Fig. 1D) is not yet feasible, *E*_F_ alignment can still be achieved *via* analysis of the two separately and carefully measured spectra, as we will discuss below.

Arguably, Method 3 can be applied for Fermi level referencing of aqueous-phase PES spectra under favorable conditions, specifically where parasitic potentials are effectively suppressed. In general, this is *explicitly* a different acquisition condition to the field-free condition required for Method 1. The work functions, e*Φ*, of the samples and the detection system usually differ, which results in a contact potential difference, Δe*Φ*, between the analyzer, the metallic reference, and/or the LJ sample in the *E*_F_-aligned case; this situation is sketched in the inset of Fig. 1D. For the meaningful application of Method 3, one instead needs to find conditions in which (1) the solution conductivity is sufficiently high to enable alignment of *E*_F_, by the exchange of charge between the solution and the grounding electrode, and (2) adequate suppression of both the streaming potential and ionization-induced sample charging is given. In this case, shifting of the liquid-phase PE features with respect to *E*_F_ in the measured spectrum can be avoided, *i.e.*, a direct relationship between the liquid and measured metallic reference spectrum can be established. Thus, after careful elimination of these influences, and the performance of two separate measurements to detect VIE_1b1(l)_ or VIE_1b1(sol)_ from the LJ and the Fermi edge from the reference metal sample, *E*_F_ referencing is in principle established. We emphasize – analogous to the gas-phase referencing approach, Method 1 – that if *extrinsic* potentials other than the aforementioned Δe*Φ* remain, *e.g.*, by insufficient compensation during the experiment, the liquid and metal spectra (*i.e.*, *measured* eKEs) are differentially affected, preventing a common energy referencing based on Method 3.

With VIE_vac,1b1(l)_ determined *via* Method 2, a comparison to VIE_EF,1b1(l)_ determined with Method 3 directly yields e*Φ*_water_ with the caveats described in Note ||. A conceptually similar procedure was previously applied by Tissot *et al.*^68^ to extract *E*_F_-referenced VIE values from static, low-vapor pressure, saturated (∼6 M) NaCl and (∼11 M) NaI aqueous solutions deposited on a gold substrate. There, the metallic and liquid features were referenced to each other under grounded conditions, with the associated approach further benefitting from being free from streaming potentials due to the static nature of the immobile liquid droplet. A value of e*Φ* was subsequently determined by biasing the sample and probing the associated isolated LET signal (see Note ¶). However, organic impurities contained in the solutions and accumulated radiation-induced sample damage may have obfuscated the true value of e*Φ*; both issues are generally negligibly small when using liquid-microjet sample-delivery methods.^37^ A subsequent attempt to determine e*Φ*_water_ using core-level LJ-PES – from 50 mM NaCl and 0.15 M butylamine aqueous solutions – was reported,^31^ albeit based on the implementation of an inadequate procedure that relied on several questionable assumptions, as detailed in ESI Section 7.†

More recently, we were made aware of a study by Ramírez,^69^ which, building on the two works mentioned above, reports VIE_1b1(l)_ and work function measurements from KCl and Zobell^70^ aqueous solutions to tune the aqueous redox potential; the reasons for and implications of implementing such a redox couple are detailed below when we present our measurements of liquid water's work function. The associated VIE_vac_, VIE_EF_, and e*Φ* values notably differ from the values reported in the present work and are elaborated on in the Results & discussion section as well as ESI Section 7.†

## Methods

Experiments were performed at four facilities, equipped with different setups. Measurements at photon energies of ∼15 eV, ∼20 eV, ∼25 eV, and ∼30 eV were conducted at the DESIRS VUV beamline^71^ of the SOLEIL synchrotron facility, Paris, using a novel LJ-PES apparatus.^72^ The same LJ-PES setup was used for He I α (= 21.218 eV), He II α (= 40.814 eV), and He II β (= 48.372 eV) measurements in our laboratory at the Fritz-Haber-Institute (FHI), Berlin, and for measurements at photon energies of ∼250 eV, ∼400 eV, and ∼950 eV at the P04 soft X-ray beamline^73^ of the PETRA III synchrotron facility (Deutsches Elektronen-Synchrotron, DESY, Hamburg). Briefly, the LJ-PES apparatus is equipped with a Scienta Omicron HiPP-3 hemispherical electron analyzer (HEA), complete μ-metal shielding, and, when not operated at a synchrotron radiation source, a VUV5k monochromatized plasma-discharge light source (He) for the laboratory experiments. Measurements at photon energies of ∼123.5 eV, ∼247 eV, ∼401 eV, ∼650 eV, and ∼867.5 eV were additionally performed using the SOL^3^PES setup^74^ at the U49-2_PGM-1 soft X-ray beamline^75^ at the BESSY II synchrotron radiation facility in Berlin.

In the low-photon-energy synchrotron experiments at SOLEIL, the light was linearly polarized perpendicular to the plane of the laboratory floor, which was the plane spanned by the LJ and light propagation axes. The analyzer collected electrons in a backward scattering geometry, forming an angle of 40° to the light polarization direction. An energy resolution of better than 3.5 meV with an on-target spot size of approximately 200 μm horizontal (in the direction of the LJ propagation) and 80 μm vertical was implemented at the LJ in these experiments. The VUV He discharge light source at FHI delivered essentially unpolarized light to the LJ *via* a minimally polarizing (<0.1%) monochromator system. The energy resolution was limited by the intrinsic width of the emission lines, 1 meV (He I) and 2 meV (He II), and the focal spot size was approximately 300 × 300 μm^2^ at the LJ. The light propagation axis of the VUV He discharge light source spanned an angle of ∼70° with respect to the photoelectron detection axis. The associated electron analyzer resolution was better than 40 meV at a pass energy of 20 eV. In the PETRA III experiments, the synchrotron beam was circularly polarized and the electron analyzer collection axis was aligned at 50° with respect to the light propagation axis (using the same analyzer geometry as in the SOLEIL experiment). The energy resolution was calculated to be 30 meV at 250 eV, 50 meV at 400 eV, 80 meV at 650 eV, and 140 meV at 950 eV with an associated focal spot size of approximately 180 μm horizontal (in the direction of the LJ propagation) and 20 μm vertical at the LJ. In the BESSY II synchrotron experiments, the light propagation axis was aligned orthogonally to the photoelectron detection axis. The U49-2_PGM-1 beamline (BESSY II) supplied linearly polarized soft X-rays with their polarization vector in the plane of the laboratory floor. The LJ and the photon beam propagated in this plane and were mutually orthogonal. The analyzer collection axis was aligned at ∼55° with respect to the synchrotron beam polarization axis. The corresponding energy resolutions were 35 meV at ∼125 eV, 70 meV at ∼250 eV, 120 meV at ∼400 eV, and 250 meV at ∼868 eV (as determined *via* gas-phase photoemission resolution calibration measurements) with a focal spot size of approximately 100 × 40 μm^2^ at the LJ.

The aqueous solutions were injected into the interaction vacuum chamber through 25–30 μm orifice diameter glass capillaries at the tip of a LJ rod assembly. The liquid flow rate was 0.5–0.8 ml min^−1^. In the EASI experiments, the temperature was stabilized to 10 °C by water-cooling the LJ rod using a recirculating chiller. In the SOL^3^PES experiments, the solutions were cooled to 4 °C within a recirculating chiller bath, prior to delivery to the vacuum chamber *via* insulating PEEK tubing. Upon injection into vacuum, the LJs exhibited a laminar flow region extending over 2–5 mm, after which Rayleigh-instabilities caused them to break up into droplets, which were ultimately frozen at a liquid nitrogen trap further downstream. The laminar-flow region was surrounded by an evaporating water gas-sheath in all cases, with rapidly-decaying local gas pressures spanning ∼10 mbar at the solution-vacuum interface and descending to the average vacuum chamber pressures with a 1/*r* distance dependence from the cylindrical LJs. The laminar region of the LJs were positioned and ionized in front of the HEA entrance apertures. The liquid-vacuum interface we refer to in the text, *i.e.*, the interface region where water's density rather smoothly decreases from its liquid bulk value to that of the gas in the immediate vicinity of the surface, is thought to evolve over a single-nm length scale.^76^ The associated solutions were prepared by dissolving NaI or NaCl (both from Sigma-Aldrich and of ≥99% purity) in highly demineralized water (conductivity ∼0.2 μS cm^−1^) and were degassed using an ultrasonic bath. Concentrations of 30–50 mM were used for all measurements performed under biased conditions. To measure liquid water spectra under field-free conditions, a conductive electrode was introduced in the electrically conductive liquid stream and electrically connected to the analyzer. In addition, at the beginning of every experimental run, the concentration of NaCl was iteratively varied in ∼10 steps to minimize the observed width of the gas-phase photoelectron peaks. Such conditions are obtained when the potential difference between the liquid jet and analyzer entrance cone is zeroed over the liquid–gas-phase sample-light-source interaction region, with field-free conditions correspondingly pertaining, at least on average. In the EASI instrument, the corresponding optimal NaCl concentration was consistently found to be 2.5 mM at a flow rate of 0.8 ml min^−1^ and a liquid jet temperature of 10 °C. The LJ rods were mounted into micrometer manipulators for high-precision alignment. The average pressures in the interaction chambers were maintained between 7 × 10^−5^ and 1 × 10^−3^ mbar using a combination of turbo-molecular pumping (∼2000 or ∼2700 l s^−1^ pumping speed for water vapor in the SOL^3^PES and EASI instruments, respectively) and two (SOL^3^PES) or three (EASI) liquid–nitrogen-filled cold traps (up to 18 000 l s^−1^ pumping speed for water vapor per trap in both instruments). The light–LJ interaction point was set at a 500–800 μm distance from the detector entrance orifice, either a 500 μm (SOL^3^PES) or 800 μm (EASI) circular differential pumping aperture. In all experiments, the LJ propagation and photoelectron detection axes were orthogonal to each other. For the experiments with the grounded LJ (field-free and streaming-potential-free measurements) all surfaces in the vicinity (at least up to 4 cm away) of the LJ-light interaction point were carefully cleaned and then coated with graphite to equalize the work function of all surfaces and prevent stray potentials: this includes the LJ rod, detector cone including the skimmer, and exit capillary of the VUV plasma-discharge light source. The glass LJ capillary was not coated. We made sure that all new glass LJ capillaries were run with water for at least a day, to passivate the inner surfaces.^28^

**Fig. 2:**
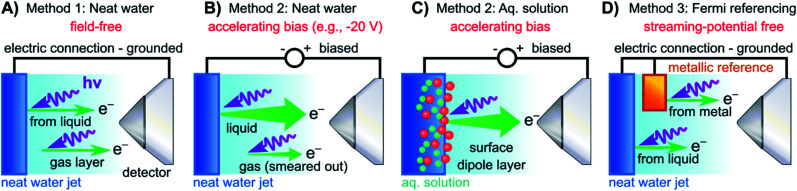
Schematic setups for the measurement procedures introduced in Fig. 1. (A) Electrically grounded (nearly) neat water LJ with a precisely tuned salt concentration to achieve a field-free condition for gas-phase referencing. (B) Negatively biased LJ used to reveal *E*_cut_ in the liquid spectrum for energy referencing; gas- and liquid-phase PE contributions are energetically separated in the field gradient. (C) Same as (B) but for an aqueous solution (here, featuring a surface-active solute). Changes in VIEs can be directly observed. (D) Similar to (A) but with the addition of a metallic reference sample held in electrical contact to and mounted within the vicinity of the LJ. The liquid water spectrum can be referenced to the Fermi edge of a metal sample under field-free conditions. Note that the metallic reference sample surface is probed separately from the LJ in the experiments reported here, and thus is not directly affected by any changes at the surface of the solution.

In both the EASI and SOL^3^PES experiments, solutions were guided through PEEK tubing all the way to the glass capillary, *i.e.*, the liquid did not come in electrical contact with the LJ rod. In the EASI experiments, the liquid flowed through a metallic grounding insert in-between the PEEK tubing prior to injection into the vacuum chamber, *i.e.*, before entering the LJ rod assembly. In the SOL^3^PES experiments, an electrical contact to the liquid was provided by an electrically insulated platinum disc inside the jet rod just before the glass capillary. This disc was connected *via* an insulated wire to an external electrical feedthrough. Both methods facilitated either the electrical grounding of the liquid to the same potential as the electron analyzer *via* a bridge cable or the deliberate application of a bias voltage to the liquid with respect to the analyzer. We emphasize that this biased the liquid solutions directly, and no external electrodes were used. Identical results were obtained with the two LJ rods. The bias voltages were applied using highly stable Rohde & Schwarz HMP4030 voltage sources. A sketch illustrating the LJ-PES experiment for a grounded and negatively biased water jet is presented in Fig. 2A and B (neat water)/2C (aqueous solution), respectively.

For the Fermi-level measurements, we utilized two metallic reference samples. Firstly, a gold wire in good electrical contact and in close proximity to the LJ (expelled by the aforementioned glass capillary nozzle) was implemented. Alternatively, a grounded platinum–iridium (PtIr) disc was used instead of the glass LJ nozzle to expel the liquid through a metallic pinhole. The PtIr disc was thus in direct electrical contact with the liquid expelled as a LJ, similar to the original LJ-PES setup utilized in ref. 4. In the SOL^3^PES experiments, both the liquid nozzle and the gold wire were mounted together on the same manipulator assembly and were moved in unison. The metal spectrum was measured with the LJ running after slightly relocating the whole assembly to bring the gold wire, instead of the LJ, into the synchrotron and detector foci. The EASI setup instead featured a retractable gold wire on a different port. A schematic of the PES measurement from a LJ in electric contact with a grounded gold target is presented in Fig. 2D. The PtIr disc was exposed to ionizing radiation through a cutout in the disc mount that was aligned towards the detector orifice; the disc was brought into the light source focus by slightly moving the rod assembly. All methods yielded the same energetic position of the Fermi level with better than 0.03 eV precision, and no changes in the Fermi-level position were detected when running different solutions.

## Results and discussion

### The accurate lowest VIE of liquid water, VIE_vac,1b1_

**Fig. 3:**
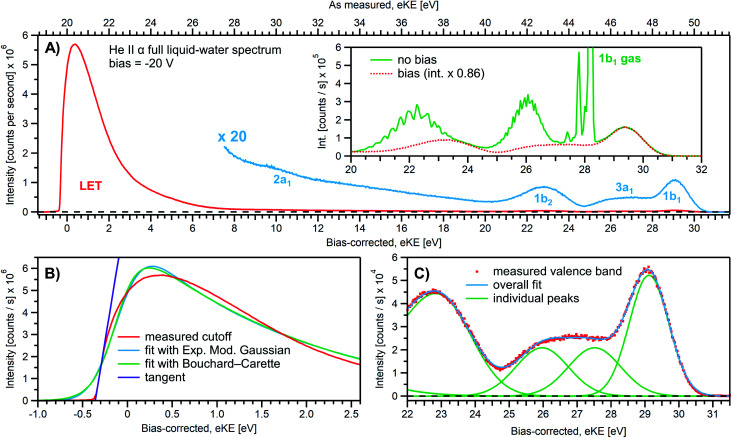
(A) A representative PE spectrum of liquid water (with 50 mM NaCl added), measured with a monochromatized He II α emission light source (*hν* = 40.814 eV). Exemplary associated electron count-rates are presented, as reported by the analyzer measurement software. Note that the count rate calibration is that provided by the analyzer manufacturer, which has not been verified under the acquisition conditions implemented here, and correspondingly should be considered a coarse guide to the overall experimental acquisition conditions only. A bias voltage of −20 V was applied to separate the liquid- and gas-phase contributions as well as to expose the low-KE tail (LET) region. The as-measured eKE is shown on the top *x*-axis in (A), with the bias-corrected scale shown on the lower *x*-axis. The same spectrum with the intensity multiplied by 20 shows the full valence band of water. The inset compares the valence region with and without an applied bias, exposing the gas-phase contribution. (B) A close-up of the cutoff region with three analysis methods applied as described in the main body of the text. The bias-corrected *x*-axis scale is plotted and the residual gas-phase contribution has been subtracted. (C) A close-up on the valence spectral region with a cumulative Gaussian fit to all ionization peaks/molecular orbital contributions, also plotted on the bias-corrected *x*-axis scale. Only the three highest energy orbitals are visible here.

We first present results obtained with the measurement schemes introduced in Fig. 1B and 2B, *i.e.*, energy referencing Method 2 introduced above. Fig. 3A shows an exemplary liquid water jet full PES spectrum in red, ranging from *E*_cut(s)_ to the eKE maximum, recorded with a 40.814 eV (He II α) photon energy and an applied bias voltage of −20 V. eKEs are presented as recorded by the spectrometer and under the influence of the applied bias on the top abscissa, *i.e.*, the quantity measured in the experiment. On the bottom abscissa, we plot the eKE scale with 20 eV subtracted to compensate for the applied sample bias. *E*_cut(s)_ is found at slightly smaller energies than zero eKE when the −20 eV compensation is applied. In general, the bias voltage is slightly reduced (here by about ∼2%) due to internal resistances between the voltage source and the liquid surface inside the vacuum chamber (for example, see Fig. SI-3†). However, the exact cutoff position can vary widely, as the precise KE scale depends on the particular experimental conditions, including the aforementioned residual resistance, LJ flowrate, electrolyte concentration, ionizing photon flux *etc.* Importantly, the absolute energetic position of *E*_cut(s)_ or any valence features in the spectrum is of no concern for our method; we specifically aim to determine energetic separations here, Δ*E*_w_ in Fig. 1B, which are not affected by the effectively applied bias voltage or any other extrinsic potential. The bias must, however, be large enough to separate *E*_cut(s)_ from *E*_cut(A)_ (where the former may otherwise be obscured by the latter, as illustrated in Fig. 1B), and be stable on the energetic scale of the eKE measurement precision and the timescale of the experiment. Whether the measured LET curve accurately reflects the true shape and intensity of the nascent electron distribution emitted by the liquid sample with respect to the characteristic valence water PES signal intensities (commonly attributed to 1b_1_, 3a_1_, 1b_2_, and 2a_1_ orbital ionization and shown in blue in the ×20 enlarged region of the spectrum), cannot be answered here. Such a determination requires careful and technically demanding calibration of the HEA transmission under the adopted conditions.‡‡‡‡The transmission function of the HEA generally influences the relative signal intensities over larger energy ranges, and especially at very small eKEs, the electron signal is distorted as slow electrons are particularly affected by stray fields (which is another reason to apply an accelerating bias). This makes it difficult to compare exact relative intensities over an energy range larger than about 30–40 eV, something which is beyond the scope of the findings presented here. Any feature within a smaller energy window, such as the valence band region or the cutoff region can be separately analyzed without further correction, since the transmission function will vary minimally over such a small energy range (assuming the cutoff electrons are sufficiently accelerated by an applied bias). A particularly important aspect is the potential effect of the analyzer electron transmission function on the LET shape upon application of a bias voltage. We find, however, that this effect has a negligibly small impact on the value of the extracted absolute VIE values, as detailed in Fig. SI-3.

Under the −20 V bias conditions employed here in order to utilize Method 2 (see Fig. 1B), most of the gas-phase water contributions are spread out over an energy range which lies below the LET of liquid water. The remaining small tail residing below the LET – accounting for less than 0.5% of the signal, depending on the bias setting and size of the ionizing light spot – has been subtracted from the data shown in Fig. 3 (note that the small signal tail below the sample cutoff feature will generally also have a secondary electron contribution created within the detection system, although modern HEAs adopt measures to minimize such parasitic signals as much as possible). For reference, the inset of Fig. 3A shows the 20–32 eV region of the valence spectrum for the grounded water jet (in green). The unbiased spectrum exhibits simultaneous gas- and liquid-phase contributions, as commonly reported in the LJ literature^4,6^ and somewhat enhanced here due to the relatively large focal spot size of the utilized VUV He discharge light source.

It is of interest to discuss the unbiased spectrum (inset of Fig. 3A) in detail. It exhibits sharp vibrationally resolved gas-phase peaks, which are generally not observed in LJ-PES experiments. Sharp spectra of gaseous molecules are readily obtained with our setups if measurements are made without the LJ installed (see, *e.g.*, Fig. SI-2B,† where the gaseous 1b_1_ HOMO ionization peak was measured by flowing gaseous water into the vacuum chamber). In that case we are not concerned with any disturbing electric fields. However, in the presence of the LJ, and with associated intrinsic and extrinsic potentials and a potential gradient acting between the LJ and the analyzer, photoelectrons from the gaseous species are accelerated differently depending on their spatial point of origin, and thus the gas-phase spectrum is inevitably broadened. In other words, a sharp gas-phase spectrum measured from water molecules evaporating from the LJ is a good indicator of a vanishing electric field in the experiments that use the relatively large focal spot of our VUV He discharge light source (300 μm beam diameter). Such a field-free condition is a very useful sensor that will be exploited in the present work. A point of caution, however, is that the ‘sharpness’ or broadening of gaseous PE features in the presence of extrinsic fields distinctively depends on experimental parameters like the spot size of the light source or experimental resolution, and is not a universal indicator of field-free conditions.§§§§A tightly focused ionizing beam – such as those provided by synchrotron or laser light sources – primarily probes gaseous molecules in the immediate vicinity of the LJ surface. The associated spectra may be only mildly energetically broadened in a field gradient and the relatively low potential difference spanning the probed volume. Consequently, associated measurements may present an apparently sharp gas-phase PE signal, despite the presence of an extrinsic field gradient between the sample and electron analyzer. The liquid spectrum measured with the −20 V bias applied is also shown in the inset of Fig. 3A (red dots), negatively shifted by the bias potential for comparison. Under these experimental conditions, an essentially pure liquid water spectrum is obtained with the gas-phase contribution shifted out of the detected energy range, as explained earlier in the manuscript in the context of Fig. 1. Note that due to experimental-geometry-dependent differences in the relative intensities of the gas *versus* liquid phase valence ionization features, the energetic positions of the liquid-phase peaks can be easily misidentified in the absence of the applied bias. Different apparent liquid peak heights in the biased and unbiased cases reflect the fact that only the 1b_1_ gas- and liquid-phase ionization signal contributions are well separated spectrally, while for all other valence ionization channels, the two contributions overlap.^4^

We next discuss the accurate determination of *E*_cut(s)_ and the position of liquid water's lowest VIE. For the former we analyze the spectral cutoff region and the LET, presented in Fig. 3B. As in Fig. 3A, the measured curve is shown in red. The purple line is the tangent extracted at the low KE inflection point, which is determined from the first derivative of the LET spectrum. The tangent intersection with the *x*-axis determines *E*_cut(s)_, a standard procedure in solid-state PE spectroscopy (for example, see ref. 77–82) that is correspondingly adopted here. The associated protocol, as well as alternative approaches to defining *E*_cut_, are described in ESI Section 5 and illustrated in Fig. SI-4.† For comparison, we apply two additional fit functions to the data shown in Fig. 3B, the Exponentially Modified Gaussian (EMG, blue curve) distribution as originally used by Faubel and co-workers to model the liquid-phase LET curves,^36^ and the distribution applied by Bouchard and Carette (green curve) as originally introduced for the description of the LET in semiconductors.^83^ Both of these distributions were previously adopted in the analysis of PES spectra from a stationary droplet of saturated NaCl and NaI solutions.^68^ However, neither of the two functions yield appropriate fits to the narrower experimental LET curves measured in the present work with a LJ sample, unlike in ref. 68, supporting the associated authors' conclusion that their LET is affected by considerable surfactant impurities. Such problems are clearly avoided with a flowing and replenishing LJ, where an intrinsically sharp cutoff spectral region can be accurately measured from a liquid water sample, a similar observation to that reported in ref. 31. We note that the cutoff position extracted through fitting one of the aforementioned functions, or an alternative simple linear fit, often depends on the user-selected fit range, whereas a derivative-based method (like the conventional tangent approach favored here) is purely determined by the data, with no free parameters. Using the tangent method, the directly measured *E*_cut_ value in our example is determined to be 19.64 ± 0.02 eV (notably including the bias-induced eKE offset; compare to the top axis in Fig. 3A). Again, the fact that this value is smaller than the bias applied at the power supply (−20.000 ± 0.015 V) is primarily assigned to a residual electrical resistance within the LJ, which has no relevance for our method, as outlined above and further discussed below.

In order to determine the position of liquid water's lowest ionization energy, VIE_vac,1b1(l)_ (pertaining to the 1b_1_ peak maximum), we fit the valence PES spectrum in accordance with the existing literature, with two Gaussians for the well-established, split second ionization threshold feature, the 3a_1_ upper and lower peaks, and a single Gaussian for the other ionization features, the 1b_1_ and 1b_2_ peaks.^4,27^ Common heights and widths of the split, second VIE (3a_1_ orbital components) were implemented for spectra recorded with sufficiently high photon energy and bias applied, *i.e.*, in spectra where those peaks were found to be undistorted (such as that shown in Fig. 3A). No other constraints were imposed on the fits. For spectra with distorted peaks and elevated inelastic-scattering background, *i.e*., spectra recorded with photon energies less than ∼20 eV, this fit procedure was not applicable (see the next paragraph). The respective fits to the Fig. 3A data, here including the lowest four water (1b_1_, two-component 3a_1_, and 1b_2_ frontier orbital) ionization contributions, are displayed in Fig. 3C. Again, the red symbols show the measured spectrum, while the green curves are the individual Gaussian fit components, and the blue curve is the cumulative fit. The lowest VIE (1b_1_) peak is centered at 49.12 ± 0.01 eV KE, on the as-measured KE scale (Fig. 3A top axis). Here and elsewhere in the manuscript, the eKE peak errors were taken directly from the least-squares fitting outputs and represent one standard deviation with respect to the determined peak positions. Together with the known photon energy, *hν* = 40.814 ± 0.002 eV, we find VIE_vac,1b1(l)_ = *hν* − eKE_1b1(l)_ + *E*_cut_ = 40.814 ± 0.002–49.12 ± 0.01 eV + 19.64 ± 0.02 eV = 11.33 ± 0.02 eV.

**Fig. 4:**
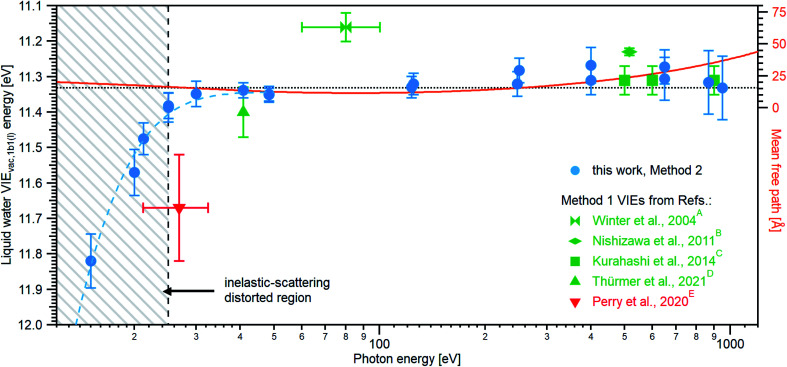
An overview of the determined VIE_vac,1b1(l)_ values as a function of photon energy. The green squares and green triangle show results obtained with the gas-phase referencing method, Method 1, where the field-free condition was achieved by carefully compensating for all potentials with a specific salt concentration: (A) from ref. 4, (B) from ref. 27, (C) from ref. 28 (where the used photon energies have been confirmed by the authors^93^), and (D) from ref. 84. The value (E) associated with the red triangle was instead obtained by applying a compensation bias voltage between the detection system and LJ to achieve a field-free condition.^29^ The values determined in this work, using Method 2, are shown as blue circles. Note that the VIE_vac,1b1(l)_ values *seemingly* shift to higher values at lower photon energies, which corresponds to low eKEs for the lowest ionization energy, 1b_1_, photoelectrons (blue dashed line in the gray hatched area). This is, however, an artifact arising from increased inelastic electron scattering at low eKEs. The averaged, nascent VIE or binding-energy value – minimally affected by electronic scattering effects – is marked with the black dashed line. Error bars show the confidence interval as reported in the studies/resulting from the analysis of our data. The electron mean free path from ref. 85 is shown as a guide to the eye in orange and on the scale to the right. While we cannot distinguish any depth dependence to VIE_vac,1b1(l)_ with the current error bars, the possibility of slight changes in VIE_vac_ with depth are discussed in the text.

Results from analogous analyses of water PES spectra measured at photon energies between ∼15 eV and ∼950 eV are shown in Table 1, and plotted in Fig. 4 (blue circles). The respective PES spectra are shown in Fig. SI-5 of the ESI.† With sufficiently high photon energies, an analogous energy referencing can be applied to the O 1s core-orbital ionization features. Although less accurate than the VIE_vac,1b1(l)_ values for the reasons we discuss below, we extract an average VIE_vac,O1s(l)_ = 538.10 ± 0.05 eV for a ∼650 eV photon energy, 538.07 ± 0.07 eV for 867.29 eV, and 538.04 ± 0.08 eV for 950.06 eV, all of which are in excellent agreement with the previous report of 538.1 eV, with an implied uncertainty of ± 0.1 eV.^55^ The error bars and error values respectively shown in Fig. 4 and reported in Table 1, as well as elsewhere in the manuscript, are the cumulative result of all error sources (calculated using standard error propagation procedures), with errors other than those arising from the peak fits being the error of the photon energy determination, error in determining the cutoff energy, and error associated with the bias-voltage shift compensation, if applied.

**Table 1:** VIE_vac,1b1(l)_ and VIE_vac,O1s(l)_ values of the liquid water valence 1b_1_ band and O 1s core-level peaks, respectively. The values were extracted from the spectra measured at different photon energies using the absolute referencing analysis method, Method 2. These values represent the averages of all measurements performed at the respective photon energy. The values in bold font are deemed to be essentially free of electron scattering based distortions of the measured VIE_vac_ values, while still being minimally affected by spectral distortions associated with the applied bias. The VIE_vac_ values shown in bold font can alternatively be referenced to the Fermi level, VIE_EF_. Such values can be ascertained by subtracting the work function of liquid water, e*Φ*_water_, determined here from the VIE_vac_ values. See the main body of the text for further details

Measured at	*hν* (eV)		VIE_vac,1b1(l)_ (eV)		VIE_vac,O1s(l)_ (eV)	
DESIRS, SOLEIL	15.00	±0.03	11.82	±0.08		
DESIRS, SOLEIL	19.99	±0.03	11.58	±0.07		
Laboratory, FHI Berlin	21.218	±0.001	11.48	±0.05		
DESIRS, SOLEIL	24.98	±0.03	11.38	±0.04		
DESIRS, SOLEIL	**29.97**	**±0.030**	**11.35**	**±0.04**		
Laboratory, FHI Berlin	**40.81**4	**±0.001**	**11.34**	**±0.03**		
Laboratory, FHI Berlin	**48.372**	**±0.001**	**11.35**	**±0.03**		
U49-2_PGM-1, BESSY II	**123.464**	**±0.004**	**11.33**	**±0.03**		
U49-2_PGM-1, BESSY II	**246.927**	**±0.005**	**11.32**	**±0.04**		
P04, PETRA III	**249.99**	**±0.02**	**11.28**	**±0.04**		
P04, PETRA III	**400.01**	**±0.03**	**11.31**	**±0.04**		
U49-2_PGM-1, BESSY II	**400.868**	**±0.004**	**11.27**	**±0.05**		
P04, PETRA III	650.03	±0.03	11.27	±0.05	538.08	±0.05
U49-2_PGM-1, BESSY II	649.67	±0.03	11.31	±0.06	538.13	±0.05
U49-2_PGM-1, BESSY II	867.29	±0.01	11.32	±0.09	538.07	±0.07
P04, PETRA III	950.06	±0.03	11.33	±0.09	538.04	±0.08

We make three major observations from the overall photon-energy-dependent VIE_vac,1b1(l)_ data shown in Fig. 4: (i) over the large photon energy range spanning 30–400 eV, we extract VIE_vac,1b1(l)_ values between 11.31 – 11.34 eV (associated with our minimum error VIE_vac_ determinations, see Table 1), (ii) for photon energies ≤30 eV, we observe an apparent significant steady increase of VIE_vac,1b1(l)_ values (accompanied by increasing error bars), and (iii) the data indicate a trend towards slightly lower VIE_vac,1b1(l)_ values for photon energies up to ∼650 eV. We start by commenting on the larger error bars determined at high photon energies, which is one aspect of point (i). At higher soft X-ray energies, a lower overall photon flux is often combined with a rapidly decreasing photoionization cross-section, requiring increased signal integration times, increasing the risk of time-dependent changes of the acquisition conditions, or compromises in the implemented acquisition settings (resolution *etc.*) needed to record sufficiently high signal-to-noise ratio data. Additionally, photon energies must be determined under the implemented experimental conditions, with highly precise photon energy calibrations required when higher photon energies are used. Such processes require utmost care and still generally result in photon energy and peak position determinations with higher absolute errors when compared to lower-photon-energy measurements. A detailed discussion of the challenges involved in accurate photon energy calibration can be found in ESI Section 3.† Another important effect to consider is the impact of the bias voltage on the detection system. A bias of several tens of volts is in effect a disturbance of the precisely tuned electron optics of a hemispherical energy (and for that matter any alternative electron) analyzer. Indeed, investigating the change in eKEs measured with our HEA systems, we find that VIE values for measurements of large eKEs can be slightly affected by the bias, depending on the bias voltage and initial kinetic energy value of the photoelectron. Specifically, it was determined that eKE values are altered by 0.015–0.035% at a bias of −64 V, depending on experimental conditions and geometric details. Fig. SI-6† showcases this effect by plotting the measured VIE_vac,1b1(l)_ dependence on the applied bias for exemplary measurements of the lowest-energy VIE at a photon energy of ∼123.5 eV. While this effect is barely noticeable at smaller photon energies, it can become detrimental to measurements at very high photon energies, resulting in several 100 meV deviations if not properly corrected for. We have corrected all values recorded above a 100 eV photon energy by either measuring the bias-voltage dependent peak-positions directly or by additionally measuring the spectrum of the same PE feature using the residual second harmonic of the beamline and comparing it to the independently calibrated photon energy used in the measurement. This yields a correction factor for the VIE_vac_ values (see ESI Section 6† for details). Finally, we note that, even without such bias-voltage induced shifts, the KE-linearity of the utilized spectrometer may be a concern when the eKEs of the measured features are far apart. In our measurements, we estimate a maximal error of ∼18 meV for the 950 eV measurements. If very high energy accuracy is required, then the linearity of the spectrometer eKE scale should be energy-calibrated, *e.g.*, by measuring known gas lines over a broad range of eKEs.

The apparent increase of VIE_vac,1b1(l)_ values (point (ii)) for the lower photon energies is an *artifact* caused by a change of electron scattering cross-sections and ionization mechanisms when tending towards lower electron KEs. For the corresponding eKEs, below ∼18 eV, the direct photoelectrons experience such severe scattering that the nascent photoelectron peak position cannot be reliably extracted.^30^ However, we deliberately include these misleading values in Fig. 4 to highlight to the reader that utmost care must be taken when trying to determine any meaningful energy in this regime. Solely applying an energy referencing scheme, be it Methods 1 or 2, without consideration of possible energy shifts due to electron scattering, will inevitably lead to erroneous results. We note that the full fit of all valence ionization features is not possible for spectra measured below 30 eV photon energies since spectral features have been considerably distorted by scattering. Accordingly, a simpler fit extracting only the lowest-ionization-energy liquid-water peak position was instead employed within that photon energy range.

**Fig. 5:**
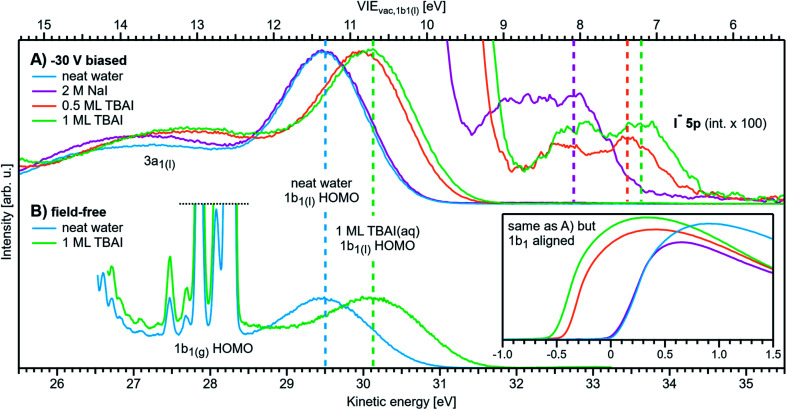
Changes in VIE_vac,1b1_ for representative aqueous solutions, both with an applied bias and a grounded jet. All spectra were recorded with He II α emission (*hν* = 40.814 eV). (A) Spectra measured with a bias voltage of −30 V. Each cutoff position was then aligned to eKE = 0 eV, which immediately visualizes VIE_vac_ changes as shifts of the liquid 1b_1_ HOMO position; the top axis shows the corresponding VIE_vac,1b1(l)_ energy scale. The bottom-right inset shows the same spectra aligned to the 1b_1_ HOMO position, which instead show a shift in the cutoff position; both presentations are equivalent. Neat water serves as a reference position (blue line; about 50 mM NaCl was added here, but the precise value is irrelevant for this method). All spectra are normalized to the same 1b_1_ peak height. The spectra are shown multiplied by a factor of 100 (and smoothed with a 5-point boxcar averaging) to reveal the I^−^ 5p solute feature to the top-right. The position of the 5p_3/2_ peak is marked with a dashed line in each case. (B) Spectra measured with a grounded jet. The salt concentration for the (nearly) neat water spectrum (blue line) was precisely tuned to achieve field-free conditions (2.5 mM NaCl was optimal here). The spectra are aligned so that the 1b_1_ position of neat water is matched with (A). The same shift is observed with 1 monolayer (ML) TBAI (green line) as in A, which shows the equivalence of Methods 1 and 2. Here, TBAI aqueous solution serves as a special case, where the field-free condition is preserved even for the solution, which makes a direct comparison possible in the first place. In general, the solutions and delivery conditions generate non-zero extrinsic and intrinsic potentials which impose an unknown additional energy shift to the liquid spectra.

From here on, we will restrict our discussion to the meaningful photon energies at and above ∼30 eV. As shown in Fig. 4, and relating to point (iii) above, the precisely measured VIE_vac,1b1(l)_ value determined using Method 2 in the present work is 11.33 ± 0.03 eV, which is the mean value based on the bold entries in Table 1 (corresponding to the plateau, *i.e.*, energies higher than ∼30 eV but excluding the results at 650 eV photon energies and above). The new value is in very good agreement with previous values reported by Kurahashi *et al.*^28^ (green squares in Fig. 4) obtained using soft X-ray photon energies and the traditional Method 1 procedure, depicted in Fig. 1A. This implies that in the experiments of Kurahashi *et al.* all extrinsic surface potentials including electrokinetic charging have been accurately compensated. Indeed, as further discussed below, our own carefully implemented field-free measurements based on energy referencing Method 1 allows us to extract fully consistent VIE_vac,1b1(l)_ values of 11.39 ± 0.08 eV at a 40.814 eV photon energy (see Fig. 5B). Comparison of our Method 1 and Method 2 results with the Method 1 results of Kurahashi *et al.*^28^ and Thürmer *et al.*^84^ accordingly indicates that any photon energy dependence of VIE_vac,1b1(l)_ is rather small (related to point (iii) above). These comparisons also suggest that any effect of an intrinsic liquid-water surface-dipole potential is negligibly small or can be adequately compensated by implementing a specific electrolyte concentration that engenders field-free conditions, at least with a cylindrical liquid-microjet source. That is, in our implemented measurement geometry, any differences between *E*^loc^_v_ in the vicinity of (nearly) neat liquid water and *E*_v_^*∞*^ seem to be below our detection limit. Considering the maximum uncertainty with which *E*_cut_ is defined in our high energy resolution data (see ESI Section 5†) and stressing that direct experimental measurements of the interfacial dipole potential, *χ*^d^, have yet to be reported, our error bars support a <50 meV value of *χ*^d^, in agreement with previous experimental inferences.^57,58^ On a related note, assuming a negligible value of *χ*^d^, the consistency of our Method 2 and properly recorded Method 1 results reinforces the use of the tangent approach to determine *E*_cut_ from an appropriately recorded LET spectrum. Were we to adopt the inflection point of the LET curve as the *E*_cut_ value instead of the tangent intersection point with the *x*-axis, we would determine just 30–100 meV higher VIE_vac,1b1(l)_ values (again see ESI Section 5†). Focusing on our high energy resolution results recorded between 40.814 eV and ∼401 eV, these offsets are limited to 30–60 meV. Thus, adopting the alternative and non-standard inflection point *E*_cut_ definition, would result in average and upper limit values of VIE_vac,1b1(l)_ of 11.38 ± 0.03 eV and VIE_vac,1b1(l)_ of 11.41 ± 0.03 eV, respectively.

Our VIE_vac,1b1(l)_ results clearly disagree with the most recently reported value from Perry *et al.*,^29^ 11.67 ± 0.15 eV (shown in red in Fig. 4). These results were based on Method 1 but were extracted by applying a small (+0.6 V) compensating bias between the jet and time-of-flight electron analyzer, under conditions where the amount of salt was not adjusted to compensate electrokinetic charging. In contrast to the originally implemented variant of Method 1, this biasing procedure seemingly has the benefit of enabling liquid-phase PES energy referencing while lifting any constraints on the concentration or type of solute under investigation (under the proviso that the solution remains sufficiently conductive). In principle, assuming sufficient care is taken to mitigate all possible perturbing potentials with the bias and to appropriately calibrate the spectrometer, this should lead to the same final result as the electrolyte tuning Method 1 scheme. However, this is obviously not the case, and the large VIE_vac,1b1(l)_ value determined by Perry *et al.*^29^ – approximately 0.3 eV higher than all of those previously reported – probably arises from a combination of inaccurate charge compensation, additional fields caused by the applied bias, and/or the aforementioned electron scattering issues. It is difficult to quantify the relative weight of these contributions *a posteriori*. We emphasize that any attempt to compensate fields by applying a bias voltage may lead to considerable eKE offsets if not properly accounted for during calibration of the energy axis of the employed (ToF) spectrometer, as demonstrated by Nishitani *et al.*^54^ In fact, for an applied bias voltage of +0.6 V, the determined energy offset reported in ref. 54 for NaBr_(aq)_ yields 0.25 eV, which would push the result of Perry *et al.* down to 11.42 ± 0.15 eV, a value well agreeing with our results (see Fig. 4 and 5B) and those of Kurahashi *et al.*^28^ Note that the average VIE_vac,1b1(l)_ value of 11.33 ± 0.03 eV found in the present work also notably disagrees with the 11.16 ± 0.04 eV reference value (green diabolo shape) measured more than 15 years ago at intermediate 60–100 eV photon energies within the range spanned in the present study; this is the first LJ-PES reference value reported by one of the present authors.^4^ A likely reason for the offset of the original 11.16 ± 0.04 eV value is again a small effect of uncompensated electrokinetic charging at a time before a precise streaming potential characterization^28,53^ was established.

We next consider photon-energy-dependent variations of the VIE_vac,1b1(l)_ value in more detail. The present study is the first to apply a broad range of photoexcitation energies, connecting the UV to the soft X-ray regime. Naturally, it is intriguing to explore the possibility that VIE_vac,1b1(l)_ may not be exactly the same for surface water molecules and those existing deeper into the bulk solution. The probing depth into an aqueous solution is thought to be at its smallest at around 60–150 eV KE, where the electron inelastic mean free path (IMFP) curve seemingly exhibits a shallow minimum and rises towards higher energies.^66,67^ Correspondingly, deeper probing into the solution should be enabled at higher photon energies. This raises the barely addressed question whether VIE_vac,1b1(l)_ is eKE-dependent, following the eKE-dependent IMFP in water. Indeed, the observed slight variation in our extracted values – together with the values of Kurahashi *et al.*^28^ – do not exclude this possibility; the IMFP from ref. 85 is plotted as a right-hand y-scale in Fig. 4 as a guide to the eye. We note that the ∼50 meV larger VIE_vac,1b1(l)_ value computed at the aqueous interface with respect to the liquid bulk^13^ is consistent with the interfacially-sensitive 125 eV and predominantly bulk-sensitive 650 eV and higher photon energy results reported here. Unfortunately, our current error bounds do not allow us to confirm such an offset though. Based on all available data, the corresponding error bars, and the good agreement between the blue and green data points in Fig. 4 – respectively measured at the low- and high-KE side of the IMFP minimum – it is argued that the KE-dependence of VIE_vac,1b1(l)_ is indeed small, specifically less than 130 meV.

### Changes of solvent VIE & solute VIE values in aqueous solutions

Following the exact same Method 2 protocol as described above for neat water, the measurement of VIE_vac,1b1_ of an aqueous solution (denoted VIE_vac,1b1(sol)_) is straightforward. Aqueous solute VIEs (denoted VIE_vac,solute_) are also readily determined, without assumptions. Such measurements are founded on the schemes introduced in Fig. 1C and 2C.

Fig. 5A compares the neat water valence PE spectrum with that of NaI_(aq)_ at 2 M concentration and tetrabutylammonium iodide (TBAI), a surfactant, at concentrations of 12.5 mM and 25 mM. These TBAI concentrations yielded approximately one half and one full monolayer (ML) of TBA^+^ coverage at the solution surface, respectively.^86^ We note that the 25 mM TBAI concentration yields approximately the same iodide surface concentrations as obtained in 2 M NaI solutions.^86^ The photoelectron spectra, including the LET and leading valence features, were again measured with a 40.814 eV photon energy, the applied bias voltage was −30 V. The spectra are aligned such that the cutoff position, determined by the tangent method, falls at eKE = 0 eV. The bottom axis thus displays the eKEs following their traversal of the solution's surface. We emphasize once more that the measured energy position of the leading photoelectron peaks or *E*_cut_ alone has no meaning, since solutes may induce several additional potentials which can arbitrarily shift all eKEs associated with different PE features. We also re-emphasize that the effectively applied bias value is not and does not need to be precisely known. The only relevant property in Method 2 is the energetic distance (and changes of this distance) between *E*_cut_ and a peak of interest, exemplified by Δ*E*_w_ in Fig. 1B and C. The inset in Fig. 5 shows LET features of the same data as shown in Fig. 5A but instead with the water 1b_1(l)_ peaks aligned; note that this corresponds to the previously adopted and unsatisfactory practice of energy-referencing aqueous solution LJ-PES data to predetermined neat water 1b_1(l)_ VIE values. Changes in the overall spectral energy widths now appear as a shift of the cutoff position; both Fig. 5A and the inset presentations are equivalent. Adopting the cutoff spectral positions, the VIE_vac,1b1(l)_ energy scale (top axis) can now be referenced from *E*_cut_*via* the precisely known photon energy. Associated solute-induced changes in the water electronic structure are discussed first, and we later focus on the lowest solute ionization channel, *i.e.*, that attributed to the first I^−^ 5p atomic orbital which corresponds to the PE features at ∼33 eV eKE.

When switching from neat water to 2 M NaI, a small and statistically insignificant (*i.e.*, within the error bars) energy shift, accompanied with a slight broadening, of the 1b_1_ peak is observed with respect to neat water; see the purple and blue curves (Fig. 5A). This is a somewhat puzzling result, seemingly at odds with theoretical works on alkali-halide solutions, specifically reporting a larger surface propensity of iodide than the sodium cation, which implies the formation of an interfacial dipole.^76^ Interestingly, the aforementioned work by Tissot *et al.* makes a related observation. Comparing concentrated NaCl and NaI aqueous solutions, which should exhibit a very different surface potential, no differences are found in the spectra;^68^ those authors discussed the possibility of surface impurities obscuring their results. We note that the 2 M NaI concentration used here may still be below the surface-enrichment regime,^87^ and higher concentrations (>6 M) may in fact lead to a more pronounced shift. However, a concentration-dependent study is beyond the scope of this work. If simple alkali-halide salts do not alter the solution's charge equilibrium at the probed interface, and thus the position of *E*_cut_ and the valence ionization features, one must assume that inter-ionic dipoles have no net component perpendicular to the solution interface. Unfortunately, there is little data available to clarify this issue, despite multiple works attempting to quantify the interfacial density profiles of different atomic ions in aqueous solutions.^38,76,88,89^ In this context, some of the authors have recently reported that concentrated electrolytes, despite changing the electronic structure of water, do not appear to lead to any significant relative energy shifts between different valence photoelectron peaks.^7^ Rather, the lowest-energy ionization peak (1b_1_) slightly broadens, with an accompanied apparent narrowing or energy-gap reduction of the split, second ionization feature (3a_1_), the latter being the more notable spectral change. Both of these behaviors are confirmed in the present data shown in Fig. 5A.

Compared to the NaI results, the TBAI aqueous solutions behave very differently, shifting water's valence electronic structure with respect to *E*^loc^_v_, as reflected in the higher measured kinetic energies (green and orange curves). This energy shift is approximately 630 meV, judged from the change of the neat water 1b_1_ peak position, in the case of a full ML of TBA^+^ (compare the green and blue spectra). A coverage of 0.5 ML leads to a smaller shift of about 530 meV (orange spectrum). We thus find average VIE_vac,1b1(TBAI)_ values of 10.80 ± 0.05 eV (0.5 ML) and 10.70 ± 0.05 eV (1.0 ML), which are both found to be considerably smaller than VIE_vac,1b1(l)_. This large decrease in VIE could have various causes: (1) resulting from changes of the intrinsic (bulk) electronic structure of the solution (as shown for NaI), (2) a change of the intrinsic electronic structure and associated charge equilibrium at the solution-vacuum interface (*i.e.*, a relative change in the positions of water's electronic bands with respect to a fixed value of *E*^loc^_v_), or (3) a change in the net aqueous surface-dipole potential and associated value of *E*^loc^_v_. A change of the bulk-water electronic structure would be hardly expected for this surface-active molecule. However, we may have to consider the possibility of changes of the aqueous electronic structure at the liquid–vacuum interface. Still, such an effect would need to be distinguished from the two other interfacial contributions, requiring establishment of a common and ideally ion-depth-invariant reference level for the two solutions. The Fermi level should be well-suited to this task and can be indirectly measured using the experimental procedure discussed in the context of Fig. 1D. However, before discussing such a referencing procedure in detail, we consider the iodide solute signal, as measured with respect to *E*^loc^_v_, which is also visible in the spectral range displayed in Fig. 5.

Iodide photoemission gives rise to the small I^−^_(aq)_ 5p doublet features (multiplied here by a factor of 100) occurring in the 32.0–34.4 eV KE region in Fig. 5. Applying a 2-Gaussian fit procedure, we determine the respective peak positions at eKEs of ∼33.6 eV (I^−^ 5p_3/2_) and ∼32.7 eV (I^−^ 5p_1/2_) in the case of a 1 ML TBAI_(aq)_ solution. Slightly lower eKEs of ∼33.4 eV and ∼32.5 eV are determined for a 0.5 ML TBAI_(aq)_ solution. This corresponds to VIE_I5p3/2_ = 7.20 ± 0.1 eV/VIE_I5p1/2_ = 8.11 ± 0.1 eV for the 1 ML and VIE_I5p3/2_ = 7.38 ± 0.1 eV/VIE_I5p1/2_ = 8.30 ± 0.1 eV for the 0.5 ML cases, respectively. In contrast, for a 2 M NaI_(aq)_ solution we find an eKE of ∼32.7 eV (I^−^ 5p_3/2_) and ∼31.8 eV (I^−^ 5p_1/2_), corresponding to VIE_I5p3/2_ = 8.08 ± 0.1 eV/VIE_I5p1/2_ = 8.90 ± 0.1 eV; the latter is in excellent agreement with our earlier work.^2^ An important finding from Fig. 5A is, therefore, that the iodide 5p ionization energy is considerably larger in the NaI aqueous solution as compared to the TBAI solution. We note that the observed effect would have been much smaller if we had used Method 1, where only the VIE_vac,1b1(l)_ − VIE_I5p_ energy distance would be accessed but not the change of VIE_vac,1b1_. While this energy separation is indeed different by about ∼0.1 eV between 0.5 ML and 1.0 ML TBAI and about ∼0.25 eV between 2 M NaI and 1 ML TBAI (as could have been observed *via* Method 1), the true change of VIE_I5p_ as determined with Method 2 would remain inaccessible. Notably, a previous study^90^ used gas-phase water features as an energy reference for 0.04 m TBAI_(aq)_ solution PES, and thus circumvented the liquid 1b_1_ VIE altogether, arriving at rather accurate VIE_I5p3/2_ = 7.6 eV and VIE_I5p1/2_ = 8.4 eV values, albeit with a potentially huge margin of error due to unknown and uncompensated extrinsic potentials. Specifically, for NaI_(aq)_, the energetic separation of water's lowest ionization energy 1b_1_ peak to the I^−^ 5p_3/2_ peak is 3.36 ± 0.05 eV, while for the 5p_1/2_ peak it is 2.41 ± 0.05 eV, in excellent agreement with previous reports.^28,68,87^Fig. 5 also shows that VIE_vac,1b1(TBAI)_ is slightly smaller in the case of 1.0 ML TBAI coverage in comparison to 0.5 ML coverage. However, the associated energy difference is smaller than the respective changes in the VIE_I5p_ energies. For 1.0 ML TBAI solutions, we see a ∼0.25 eV increase in the water 1b_1_ to I^−^ peak separations in comparison to the 2 M NaI case. This corresponds to 3.60 ± 0.05 eV and 2.65 ± 0.05 eV separations of the 5p_3/2_ and 5p_1/2_ peaks to the water 1b_1_ peak, respectively. This aspect will be considered further below.

We close this sub-section by re-connecting the results reported here to the applicability of Method 1. Fig. 5B presents additional PES spectra from neat water and 1.0 ML TBAI, now measured for grounded solutions, *i.e.*, applying Method 1. We observe the very same positions of VIE_vac,1b1(l)_ as in the upper trace, obtained with Method 2. The reason for this (perhaps surprising) quantitative agreement is that in this particular Method 1 measurement all external fields were successfully compensated. This is true for both neat water and the TBAI solution spectra as judged by the sharp water–gas-phase features; we re-emphasize that the extrinsic fields between the sample and analyzer can only be meaningfully assessed when a sufficiently large gas volume around the LJ is probed. Establishing the necessary field-free conditions to achieve such measurements is however experimentally difficult and time-consuming. More importantly, these conditions are impossible to achieve for most aqueous solutions outside a very limiting concentration range, and only Method 2 will undoubtedly provide the correct ionization energetics. In the next section, we extend our newly applied energy referencing methodology a step further, establishing a common Fermi level for neat water and a metallic reference sample that allows determination of the VIE of liquid water with respect to *E*_F_, VIE_EF,1b1(l)_. Furthermore, in combination with the Method 2 results, it provides access to the liquid water e*Φ* value, e*Φ*_water_.

### Fermi-referenced VIEs & work functions of liquid water & aqueous solutions

As argued when describing Fig. 1D and 2D, we can formally introduce Fermi-level referencing when liquid water or an aqueous solution is in electrical equilibrium with a metallic reference sample, an approach we term Method 3. As explained in the introduction, we can measure the valence spectrum from a solution and *E*_F_ from a metal in sequential experiments. But exactly what information does this provide? With the two systems in electrical contact, *E*_F_ and the bulk chemical potential of the solution and the metal are aligned. However, in the PES experiment, one measures photoelectrons from the solution or metal after they have traversed the sample–vacuum interface and different corresponding surface dipole potentials. Ideally, one would measure the Fermi edge of the metal through a thin sheet of the flowing solution, such that electrons emitted from the metal and the bulk solution would experience the same (intrinsic and extrinsic) solution-vacuum surface potential and *E*^loc^_v_. However, as of yet, this remains experimentally unfeasible.¶¶¶¶Such a measurement would be forced to deal with a further complication: the electrons from the metal would experience both the metal–solution interfacial potential and the aqueous-vacuum potential, whereas the solution phase electrons would experience the latter only. It can still be argued, however, that *E*_F_ would be equilibrated throughout this system as long as the solution was sufficiently conductive. Considering alternative methodologies for the co-determination of solution- and solid-phase electron energetics, application of the ‘dip-and pull’ PES method may seem appropriate.^96^ However, a significant associated challenge lies in achieving sufficient control over the composition and cleanliness of the solution–vacuum interface, as well as the composition of the solution bulk following solution pulling and under a significant cumulative ionizing radiation load. Despite this, it can still be argued that a Fermi-level alignment can be achieved between the LJ and metallic reference if *streaming-potential-free* conditions are engineered, *i.e.*, under the experimental conditions depicted in Fig. 1D. We define these conditions as those that preserve the intrinsic liquid solution Δe*Φ* value with respect to the analyzer, while mitigating the remaining extrinsic potentials. It is important to differentiate these conditions from the field-free alternative discussed in the context of Method 1, where the sum of *all* potentials between the sample and analyzer are compensated to zero. This point is particularly noteworthy as the establishment of field-free conditions has previously been symbolized as ‘*Φ*_str_ = 0’.^28^ In general, the optimal solution concentrations for field-free and streaming-potential-free conditions differ, offset by the magnitude of Δe*Φ* in the experiment. Only if Δe*Φ* happens to be zero (for a particular experiment) will these two conditions be simultaneously achieved (at a fixed LJ nozzle morphology, jet flow rate, and solution temperature).

**Fig. 6:**
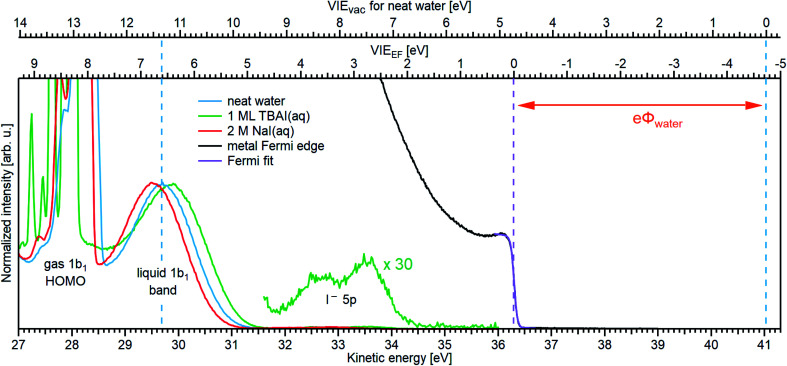
Determination of VIE_EF,1b1(l)_ for neat water (blue, with an optimal NaCl concentration of 50 mM; see the main body of the text for details) and the limitations of this method for aqueous solutions, exemplified here for 1 ML TBAI_(aq)_ (green) and 2 M NaI_(aq)_ (red) solutions. The relative energy position of liquid water's lowest energy 1b_1_ ionization feature and the Fermi edge of a metallic reference sample were separately recorded using He IIα emission (*hν* = 40.814 eV). A sample bias was not applied in either case and the bottom axis shows the as-measured kinetic energy scale of the detector. To the right, the highest eKE feature of the metal spectrum is shown in black (only the Fermi edge is visible). The position and spectral shape of the measured metal spectrum was unchanged following the introduction of the LJ and solution. The Fermi edge was fit with a Fermi function^23^ (purple line), and its position defines the zero point of the VIE_EF_ energy scale in the spectrum (lower axis scale at the top of the panel). This enables us to determine the VIE_EF,1b1(l)_ value of 6.60 ± 0.08 eV and a e*Φ*_water_ value of 4.73 ± 0.09 eV for (almost) neat water. For the 2 M NaI_(aq)_ solution, the 1b_1_ peak is shifted towards lower eKEs (higher VIE_EF_), which most likely arises from additional extrinsic fields as opposed to a real change of the aqueous electronic structure for this solution (compare to Fig. SI-7†); the VIE_vac_ values underwent insignificant changes in going from neat water and 2 M NaI_(aq)_ solutions (compare to Fig. 5). Without proper assessment of additional potentials, such as the streaming potential or surface charge, it is in principle impossible to accurately reference eKEs to *E*_F_ or judge associated changes in VIE_EF_ in this case. In the case of TBAI_(aq)_, on the other hand, the 1b_1_ shifts towards higher eKEs (lower VIE_EF_). It can be argued that this shift is caused by band-bending at the liquid interface (see text for details). Multiplying the TBAI_(aq)_ spectrum by a factor of 30 reveals the I^−^ 5p solute features around eKE ≈ 33 eV, corresponding to VIE_EF_ values of 3.80 ± 0.10 eV and 2.84 ± 0.10 eV for the 5p_1/2_ and 5p_3/2_ levels, respectively.

In the following, we briefly discuss how streaming-potential-free conditions may be established by considering the streaming current of the aqueous sample, *I*_str_, which is the source of the streaming potential, *Φ*_str_, and a less ambiguous quantity. *I*_str_ has been measured independently from *Φ*_str_, where it was shown that the aqueous streaming current is minimized at roughly 50 mM alkali halide salt concentrations, with a LJ flow rate of 0.5 ml min^−1^, and with similar LJ nozzle orifices as implemented here.^28,53^ Accordingly, a 50 mM NaI salt concentration provides a basis for our Fermi-referencing measurements.||||||||Note that an optimal concentration of 30 mM (at a flow rate of 0.5 ml min^−1^ and at room temperature) has also been reported to establish field-free conditions,^28^ which however depends on experimental parameters like the size and sign of the sample-spectrometer contact potential or work function difference, Δe*Φ*. We remind the reader that our field-free conditions were established under rather different conditions with a 2.5 mM NaI concentration, a flow-rate of 0.8 ml min^−1^, and a tapered fused silica capillary nozzle with a 28 μm orifice diameter. Associated nominally streaming-potential-free liquid water PES results recorded with a photon energy of 40.814 eV are shown in Fig. 6 (blue curve). At higher eKEs, we show the related Fermi-edge spectrum of the metal reference sample (black curve) sequentially recorded under the same conditions, as sketched in Fig. 2D. Here, the liquid water jet was in operation in close proximity to a gold wire or was directly injected from a conductive PtIr disc during these measurements. An associated fit to a Fermi–Dirac distribution is also shown (purple curve), from which we obtain the Fermi-edge position at eKE_EF_ = 36.296 ± 0.005 eV. This position defines the zero of the VIE_EF_ energy and chemical potential scale (lower axis at the top of the figure), to which all liquid-water features can now be referenced. The difference between VIE_vac,1b1(l)_ and *E*_F_, as determined from our fits, corresponds to VIE_EF,1b1_ = 6.60 ± 0.08 eV. Analogous measurements were performed using 125.02 ± 0.03 eV and 649.946 ± 0.005 eV photon energies and yielded the same results.

To examine whether streaming-potential-free conditions were established when recording the liquid water data shown in Fig. 6, and the associated validity of the measured VIE_EF,1b1_ value, a series of aqueous-phase PES spectra in electrical contact with a grounded metallic reference sample were recorded as a function of salt concentration (NaI and NaCl were found to exhibit the same effects). This allowed us to track the shift of aqueous-phase PE features with respect to *E*_F_. The resulting spectra are plotted in Fig. SI-7,† with the energetic position of the Fermi level found to be fixed within 0.03 eV, regardless of the type of aqueous solution present. That is, the metallic spectrum appears to be unaffected even by relatively high extrinsic potentials at the LJ (in some cases exceeding 1 eV). One may speculate that such potentials are effectively screened and thus terminated at the metal, nullifying any field gradients in the region between the metal and the detector. However, in contrast in the liquid water case, the lowest VIE 1b_1_ feature shifts dramatically under the influence of the varying salt concentration and streaming potential, displaying the expected behavior and exhibiting a minimum VIE_EF,1b1_ value around 50–100 mM concentrations, *i.e.*, covering the concentration implemented to produce the blue curve in Fig. 6 and where *I*_str_ (and in turn *Φ*_str_) is expected to vanish.****Such a nonlinear shift in the streaming potential has been observed before. However, no explanation has been given for the high-concentration behavior.^28^ This implies that streaming-potential-free conditions have indeed been achieved in producing the liquid water data shown in Fig. 6.

Recalling our aforementioned determination of the VIE_vac_ energy scale of liquid water using Method 2 (see the upper axis above Fig. 6), we are now set to relate the vacuum and *E*_F_ energy scales to each other. Since e*Φ* is equivalent to the difference between these two energy scales, *i.e.*, between the ionization energies of any of liquid water's ionization features measured with respect to *E*^loc^_v_ and *E*_F_, we can accordingly determine e*Φ*_water_. For example, VIE_vac,1b1(l)_ – VIE_EF,1b1(l)_ = 11.33 ± 0.03–6.60 ± 0.08 eV and yields e*Φ*_water_ = 4.73 ± 0.09 eV. By extension, one can further argue in the case of neat water that if the surface dipole/outer potential is zero, near-zero, or averages to zero in our experiments, then e*Φ*_water_ ≈ *μ̄*, *i.e.*, the determined work function is equivalent to water's electrochemical potential *μ̄*, which is a generally un-measurable quantity. We again stress that without establishing streaming-potential-free conditions, arbitrary VIE_EF,1b1(l)_ and thus e*Φ*_water_ values would be recorded, depending on the strength and sign of any extrinsic potentials; as demonstrated by Fig. SI-7.† Generally, this will remain a problem whenever the metallic reference spectrum is measured separately from the solution spectrum (*i.e.*, unless the Fermi edge signature and liquid features of interest are recorded in the same spectrum, following ejection *through* the liquid surface). This issue is unfortunately somewhat obscured when the metallic reference sample is used to initially establish an alternative (but nonetheless flawed) reference, such as the analyzer work function, as proposed, *e.g.*, in ref. 31 and 69.

As a further cross-check of our VIE_EF,1b1_ and e*Φ*_water_ results, and that streaming-potential-free conditions are indeed achieved, we extract and utilize our analyzer work function, e*Φ*_A_. To achieve this, we measured PES spectra of the metallic reference sample, either directly recording the Fermi level (e*Φ*_A_ = *hν* − KE_EF_) or some other well-calibrated metal energy level such as the gold 4f level (e*Φ*_A_ = *hν* − BE_4f_ − KE_4f_). The extracted e*Φ*_A_ is an arbitrary value in itself, and only equals the analyzer work function if the measured kinetic energy, eKE_meas_, of the detection system has been precisely calibrated using known (gas-phase) reference photon and ionization energies. We briefly describe the procedure to achieve such a calibration and compare the e*Φ*_A_ result to the field-free condition, specifically assuming this corresponds to Δe*Φ* = −e*Φ*_str_. Using the kinetic energy position of the equilibrated water gas-phase 1b_1_ peak (compare to Fig. SI-2†) and the associated reference VIE value of 12.621 ± 0.008 eV,^3^ we find that the kinetic energy scale of the detector needs to be corrected by +0.224 ± 0.008 eV; note that this value depends on the pass energy setting and detector mode. This yields a corrected Fermi-edge position of eKE_EF_ = 36.520 ± 0.009 eV from which we determine e*Φ*_A_ = 4.293 ± 0.009 eV, a value approximately 0.43 eV smaller than e*Φ*_water_. It is intriguing to then compare this value to the shift in the liquid water 1b_1_ position when going from our streaming-potential-free (50 mM) to field-free conditions (2.5 mM), *i.e.*, where Δe*Φ* = −e*Φ*_str_. There we observe that the 1b_1_ peak shifts to lower eKEs (compare to Fig. SI-7†) and that the overall shift between these two concentrations matches the expected 0.43 eV. This nicely demonstrates the shift from *Φ*_str_ = 0 V conditions to Δe*Φ* = −e*Φ*_str_ conditions, that the liquid water 1b_1_ peak follows the change in potentials one-to-one, and that streaming-potential-free conditions were indeed achieved with 50 mM NaI concentrations (under our implemented conditions). Correspondingly, our values of VIE_EF,1b1_ and e*Φ*_water_ are also confirmed.

Our established experimental value of e*Φ*_water_ is found to be somewhat larger than that reported by Olivieri *et al.*,^31^ 4.65 ± 0.09 eV, who also attempted to determine e*Φ*_water_ using LJ-PES. This work extracted the value of e*Φ*_water_ from the ‘midpoint’ of the rise of the LET curve (referred to as the SEED in the ref. 31, see Note ¶) as opposed to the tangent method commonly adopted for solid-state samples and in extracting the results reported here. In our data, this *E*_cut_ determination method has been shown to result in VIE increases of several 10 meV up to ∼150 meV (depending on the energy resolution of the experiment and the associated shape of the LET). This would be directly transferred to an increase of our value of e*Φ*_water_, bringing our determination of this value further away from that reported by Olivieri *et al.* With a comparison of these and our own e*Φ*_water_ value determinations in mind, we highlight a number of methodological inconsistencies and inaccuracies in the Olivieri *et al.*^31^ study in ESI Section 7.†

Turning now to an attempted determination of *E*_F_ and e*Φ* from an aqueous solution, we recall that our auxiliary Fermi-referencing procedure, Method 3, is not applicable to, *e.g.*, the 2 M NaI solution considered in Fig. 5, as the streaming current is thought to be non-zero (see ESI Fig. 7†). Although a precise value cannot be determined in this work due to the coupling of higher salt concentrations to *Φ*_str_, we can compare VIE_EF,1b1(l)_ (*i.e.*, from water) with the respective value from Tissot *et al.*^68^ for saturated alkali-halide solutions deposited on a gold substrate. There a 0.4 eV smaller VIE_EF,1b1(sol)_ of 6.2 eV was reported. However, we note that for higher concentrations of 2 M NaI (Fig. 6) and 4 M NaI (Fig. SI-7†) the 1b_1_ peak notably shifts to higher VIE_EF_ (lower eKEs) values, *i.e.*, even further away from the reported 6.2 eV VIE_EF,1b1(sol)_ value. The shift observed in our high-concentration measurements is likely caused by a non-zero *Φ*_str_, and one can only speculate about the true VIE_EF,1b1(sol)_ value in the absence of *Φ*_str_. However, a value of 6.2 eV is deemed unlikely. We may speculate that, to some extent, this 6.2 eV determination reflects additional extrinsic surface potentials present at the interface of the concentrated solution in the Tissot *et al.* study.^68^ This is consistent with an observed ∼0.6 eV energy shift of the O 1s gas peak towards lower eKE (higher VIE) when retracting the sample,^68^ caused either by radiation-induced sample changes or accumulation of surface impurities at the non-replenishing liquid-on-solid sample.

We now return to our TBAI aqueous solution measurements, where we observed large changes in VIE_vac_. At a bulk concentration of 25 mM, the solution conductivity is sufficient to effectively apply a bias voltage of −30 V, and we can correspondingly assume alignment of *E*_F_ throughout the solution under unbiased conditions, similar to the 25–50 mM NaCl or NaI aqueous solution cases discussed above. Consequently, we can determine *E*_F_ following the same steps as for neat water. For that we reproduce the TBAI aqueous solution spectrum from Fig. 5B in Fig. 6 (green curve), and compare it to the Fermi edge spectrum from the metallic sample (black curve), fully analogous to the water experiment. As discussed above, even when measured in the presence of the running TBAI-solution jet, electrically connected to the metallic sample, the same *E*_F_ reference value is observed as for neat water. Neither *I*_str_ nor *Φ*_str_ measurements have been reported for this solute to our knowledge, and are beyond the scope of this work. However, as we argue in the following, *Φ*_str_ may in fact be immeasurably small or even zero in this particular case. Before explaining this further, we discuss the principal results from the green curves in Fig. 6. Initially assuming *Φ*_str_ ≈ 0 V, we determine that water's 1b_1_ PE peak shifts to 0.15 ± 0.11 eV lower Fermi-referenced VIE values in the TBAI_(aq)_ solution in comparison to (nearly) neat water, with VIE_EF,1b1(TBAI)_ = 6.45 ± 0.08 eV. Using the results from Method 2 we can now, analogously to the water case, determine the solution's work function: e*Φ*_TBAI_ = VIE_vac,1b1(TBAI)_ – VIE_EF,1b1(TBAI)_ = 10.70 ± 0.05–6.45 ± 0.08 eV = 4.25 ± 0.09 eV. This corresponds to a 0.48 ± 0.13 eV reduction with respect to neat water. Considering the anionic solute components of the solution, we further extract VIE_EF,I5p_ values of 3.80 ± 0.10 eV and 2.84 ± 0.10 eV for the 5p_1/2_ and 5p_3/2_ peaks of the I^−^ solute feature, respectively.

We have seen that field-free conditions are seemingly achieved for 25 mM TBAI solutions (implied by the sharp water gas-phase spectrum in Fig. 5B and 6), which must mean e*Φ*_TBAI(aq)_ − e*Φ*_A_ ≈ −*Φ*_str_. Recalling that e*Φ*_A_ = 4.293 ± 0.009 eV, it follows that e*Φ*_TBAI(aq)_ = 4.25 eV ≈ e*Φ*_A_, *i.e.*, the *E*^loc^_v_ levels at the sample and analyzer are aligned, implying *Φ*_str_ ≈ 0 V. We have also observed that *Φ*_str_ ≠ 0 V for the 2 M NaI_(aq)_ solutions, as can be seen in Fig. 6 from the offset of the spectrum towards slightly higher apparent VIE_EF,1b1_ values. However, Method 3 does not reveal whether e*Φ*_TBAI(aq)_ or *Φ*_str_ is compensating the extrinsic potential, implying that the observed shift in VIE_EF,1b1_ may come from an active *Φ*_str_ and that the field-free condition achieved here is just a coincidence. The origin of the observed energy shift, *i.e.,* the change of VIE_vac,1b1(l)_, in Fig. 5 and 6 thus remains unresolved and cannot be confirmed with the currently available experimental tools. However, we briefly discuss how a real change in VIE_EF,1b1_, *i.e.*, under the premise that *Φ*_str_ = 0 V, would be realized below.

Dissolution of a salt in water produces hydrated anions and cations, which can be viewed as ionized dopants freely moving in the aqueous solution. At the interface to vacuum this would give rise to the band bending (BB) phenomenon commonly encountered in the semiconductor literature, and illustrated in Fig. SI-1B.† In the present case, BB is argued to be induced in response to TBAI accumulation, which changes the charge distribution at the liquid − vacuum interfacial layer. Briefly, BB occurs if there is a local imbalance of charge near the surface which leads to the build-up of a local field.^26,35,91,92^ Arguably, we observe an upward BB, *i.e.*, in the direction of lower VIEs, which is caused by a depletion of the solvent's electron density near the surface. The hydrophobic TBA^+^ molecules which reside near the solution's surface are thought to draw I^−^ ions into this surface region.^86^ It can then be argued that the solvation of I^−^ reduces water's local electronic density, leading to the observed effect. Notably, the Fermi level remains fixed (the Fermi level is pinned) within the solution at its bulk value and aligned with the metal reference and analyzer, as shown in Fig. SI-1B.† Some intriguing observations support this interpretation. The 1b_1_ HOMO peak slightly broadens when moving from 50 mM NaI_(aq)_ to 25 mM TBAI_(aq)_ solutions, which may indicate that an interfacial region with a solution-depth-dependent potential energy gradient of the 1b_1_ band is probed, implying different effective 1b_1_ energies within this so-called space-charge layer. Also, the I^−^ 5p peaks are shifted the farthest, which would be plausible given that most of the iodide resides directly at the surface, where the most disturbance of the bulk equilibrium occurs. One might correspondingly ask whether the neat water surface is already subject to BB, keeping in mind that intrinsic surface BB caused by the presence of surface defect states is a common phenomenon for semiconductors.^92^ While we cannot rule out this possibility completely, it is important to note that the water surface is very different from an abruptly terminated crystal lattice, and the dynamic nature of liquid water is likely to compensate for any charge imbalance, unless such charge accumulation is forced as in the case of surface-active species such as TBAI. Thorough exploration and characterization of such effects using photon-energy- and thus solution-depth-dependent Fermi-referenced LJ-PES measurements is an associated interesting future line of research directly enabled by the work reported here.

Until now we have adopted surface-science concepts to interrogate and interpret aqueous-phase PES data, providing a useful methodological advancement to access an explicit descriptor of solution interfacial electronic properties, namely the work function *via* joint determinations of VIEs with respect to *E*^loc^_v_ and *E*_F_. In the following, we briefly discuss the impact of this accomplishment in the wider context of interfacial chemistry and electrochemical processes, in particular at the metal–electrode – electrolyte system. This very ensemble of a LJ electrically connected with a metal sample (again, see Fig. 2D) represents a single electrode immersed into an electrolyte. As we have explained above, connection of a metal to a sufficiently conductive liquid water or aqueous solution sample (both classifiable as semiconductors) yields a common Fermi level. In the case of an electrolyte containing both forms of a redox couple (representing vacant and populated energy levels within the band gap, separated by *E*_F_^15^), the redox level, *E*_redox_, can be equated to *E*_F_ in the solution and aligned with *E*_F_ of the metal.^26^ This implies that *E*_F_ of the solution shifts with charge flow across the interface until *E*_F_ = *E*_redox_, where the two energy scales for the aqueous solution and the potential scale for the electrode are connected through the theoretical value of the Fermi level of the standard hydrogen electrode. This route has been explored in a very recent LJ-PES study,^69^ determining *E*_F_*via* the aqueous-phase ferricyanide/ferrocyanide redox couple (in a Zobell^70^ solution), and reporting values of VIE_EF,1b1(l)_ = 6.94 eV and e*Φ* = 4.60 eV, both of which differ from our results for neat liquid water. Furthermore, a much larger VIE_vac,1b1(l)_ value of 11.55 eV was reported for the Zobell solution. We highlight a number of potential issues with the methodology adopted in ref. 69 in ESI Section 7,† which we believe may be responsible for the discrepancies between our and their results. We also note that most of these problems could be circumvented by rigorously applying Method 2, as presented in this work.

In a more general context, and not requiring introduction of redox couples, it will be possible to use known electrode potentials and measured Fermi levels to locate the band edges of liquid water and select aqueous solutions on the chemical potential scale.^26^ This is not only of uttermost importance for advancing our understanding of chemical reactions at electrode–electrolyte systems but it also enables future routes to develop a common interpretation of thus far seemingly disconnected quantities specific to the molecular and condensed-matter descriptions of electronic structure. One pressing example is how the band gap of liquid water conceptually connects with the molecular-physics or orbital information accessed by LJ-PES, including an experimental determination of liquid water's electron affinity.^13^

## Conclusions

Liquid microjet photoelectron spectroscopy (LJ-PES) is an indispensable experimental tool for the characterization of electronic–structure interactions in liquid water and aqueous solutions. This includes the determination of valence electron energetics, which is key to understanding chemical reactivity. And yet, the full potential of this method is just about to be exploited, entailing several important benefits, discussed in the present work. This includes the measurement of *absolute* solute and solvent energetics and the accessibility of a specific interfacial property descriptor, the work function (something that is routinely obtained in solid-state PES). Specifically, we have demonstrated the necessity of measuring the liquid-phase low-energy cutoff spectrum along with the photoelectron peak of interest. This approach has several major advantages over the formerly adopted LJ-PES energy referencing scheme and correspondingly has far-reaching implications. With the help of the cutoff energy, *E*_cut_, absolute solute and solvent energies can be robustly, accurately, and precisely measured without assumptions, no longer requiring the long-practiced and unsuitable energy referencing to the lowest-energy VIE_1b1_ of *neat* liquid water. Using the methodology introduced here, we find an average VIE_vac,1b1_ of 11.33 ± 0.03 eV (with respect to *E*^loc^_v_) for neat water, and attribute several previously measured and offset values to the effects of perturbing surface charges, with various condition-dependent potential origins. *Via* a broad photon energy dependent study of VIE_vac,1b1_, spanning the UV and a large portion of the soft X-ray range, there is a further indication of a small photon energy dependence of VIE_1b1_, although a definitive answer has to be postponed until the challenge of precisely measuring VIEs with a small error at high photon-energies can be overcome. We further demonstrated the emergent ability to measure solute-perturbed VIE_vac,1b1_ values from aqueous solutions, *i.e.*, solute-induced effects on water's electronic structure. With the same experimental approach, solute energies can be accurately measured, something which is exemplified here using aqueous iodide solutions. Extending our proposed energy referencing approach to deeper-lying electronic states, we have additionally reconfirmed and more precisely defined water's O 1s core-level binding energy, extracting a value of VIE_vac,O1s_ = 538.10 ± 0.05 eV at a ∼650 eV photon energy.

Regarding the interfacial properties of water and aqueous solutions, we have described and applied a procedure that allows the formal determination of the Fermi level of neat water and select aqueous solutions. Our approach is based on the measurement of LJ-PES spectra under conditions where the streaming potential associated with the flowing LJ has been mitigated. It further relies on the separate measurement of the Fermi edge spectrum from a metal sample in good electrical contact with the electrolyte and electron analyzer. This allowed us to accurately determine VIE_EF,1b1_ = 6.60 ± 0.08 eV. Building on this approach and the separate accurate measurement of vacuum-level-referenced VIEs (as discussed above), interface-specific aqueous-phase work functions have been extracted, including that of liquid water. Here, e*Φ*_water_ was accurately determined to be 4.73 ± 0.09 eV. Based on the collective electronic structure information accessed both with respect to *E*^loc^_v_ and *E*_F_ over the course of this study, we have carefully discussed the observed solution-specific energy shifts of the *E*_cut_ feature and/or VIE values, which have allowed us to differentiate solution work function and solute-induced (bulk) electronic structure changes. This included quantification of a nearly 0.5 eV aqueous solution e*Φ* reduction upon dissolution of a known surfactant (25 mM TBAI).

Still, our study also highlights current shortcomings in state-of-the-art liquid-phase experimental methodologies, particularly the difficulties in *E*_F_-referencing arbitrary, free-flowing aqueous solutions and determining their work functions. This primarily stems from the challenges associated with mitigating solution streaming potentials, irrespective of solute concentrations, surface dipole potentials, and the employed experimental conditions. In the particular case of liquid water, we have shown that the aforementioned limitations can be circumvented by measuring and zeroing streaming potentials, while taking advantage of liquid water's small *χ*^d^ value, or small effective *χ*^d^ value in our experimental geometry. Here, the inaccuracies of this approach have been determined to amount to less than 50 meV, notably within our VIE_EF,1b1_ and e*Φ*_water_ error ranges. However, in the case of concentrated aqueous salt solutions, such an approach could not be adopted, specifically due to the presence of unknown streaming potentials *and χ*^d^ values. To overcome these limitations, an alternative and more general *E*_F_-referencing method would need to be realized. An intriguing associated approach would be the detection of photoelectrons from a solid sample (specifically a metal) covered with a thin layer of flowing electrolyte, engendering metal- and solution-born electron collection *via* the same, generally charged liquid interface. This, however, remains a formidable challenge, particularly for PES studies aiming to resolve the microscopic (electronic) structure and chemical processes occurring at solid–solution interfaces.^65^ Irrespective of the various technical hurdles ahead, the work presented here is a major enrichment of the LJ-PES technique, enabling the general, direct, and accurate measurement of absolute electron energetics within the liquid bulk and at liquid-vacuum interfaces of aqueous solutions. Concurrently, this work brings us a step closer to bridging the gap between solid-state and liquid-phase PES, and more importantly the surface science and (photo)electrochemistry research disciplines.

## Note added in proof

We were made aware of another liquid jet X-ray photoelectron spectroscopy study of aqueous solutions concerned with cutoff- and Fermi-level energy referencing, which is currently in press.^97^ This work summarizes some of the results within ref. 69, where a number, although not all, of our associated points raised in section 7 in the ESI have been addressed.

## Data availability

The data of relevance to this study have been deposited at the following DOI: 10.5281/zenodo.5036382.

## Author contributions

S. T., B. W., and I. W. designed the experiments and, together with S. M, F. T., and with occasional assistance from C. L., performed the measurements. S. T., B. W., S. M., and I. W. analyzed the data. S. T., B. W., and I. W. wrote the manuscript and the ESI† with critical feedback from all co-authors.

## Conflicts of interest

There are no conflicts to declare.

## Supplementary Material

SC-012-D1SC01908B-s001
