# Supplementary material for: Accurate vertical ionization energy and work function determinations of liquid water and aqueous solutions
Source: Chem Sci. 2021 Jul 2;12(31):10558–82. doi: 10.1039/d1sc01908b (PMC8356740; doi:10.1039/d1sc01908b)
Supplement: SC-012-D1SC01908B-s001 [file SC-012-D1SC01908B-s001.pdf]

Electronic Supplementary Material (ESI) for Chemical Science.
This journal is © The Royal Society of Chemistry 2021

Supporting Information

Accurate Vertical Ionization Energy and Work Function Determinations of Liquid Water and Aqueous Solutions

Stephan Thürmer,[1]* Sebastian Malerz[2], Florian Trinter[2,3], Uwe Hergenhahn[2], Chin Lee,[4,5] Daniel M. Neumark,[4,5] Gerard Meijer,[2] Bernd Winter,[2]* and Iain Wilkinson[6]*

[1] *Department of Chemistry, Graduate School of Science, Kyoto University, Kitashirakawa-Oiwakecho, Sakyo-Ku, Kyoto 606-8502, Japan*

[2] *Molecular Physics Department, Fritz-Haber-Institut der Max-Planck-Gesellschaft, Faradayweg 4-6, 14195 Berlin, Germany*

[3] *Institut für Kernphysik, Goethe-Universität, Max-von-Laue-Straße 1, 60438 Frankfurt am Main, Germany*

[4] *Chemical Sciences Division, Lawrence Berkeley National Laboratory, Berkeley, CA, 94720 USA*

[5] *Department of Chemistry, University of California, Berkeley, CA, 94720 USA*

[6] *Department of Locally-Sensitive & Time-Resolved Spectroscopy, Helmholtz-Zentrum Berlin für Materialien und Energie, Hahn-Meitner-Platz 1, 14109 Berlin, Germany*

List of Symbols and Acronyms

General: subscript (l) = liquid-phase features, subscript (g) = gas-phase features, subscript (aq) = solute features in an aqueous solution, subscript (sol) = water features in presence of a solute.

Symbol / Acronym	Meaning
**PE**	photoelectron
**PES**	photoelectron spectroscopy
**HEA**	hemispherical electron energy analyzer
**ToF**	time-of-flight spectrometer
**LJ**	liquid jet
**mM / M**	millimolar / molar
**ML**	monolayer
**BB**	band-bending
**HOMO**	highest occupied molecular orbital
**LET**	low-kinetic energy tail
**SEED**	secondary electron energy distribution
**IMFP**	inelastic mean free path
**HHG**	high harmonic generation
**VUV / EUV**	vacuum-ultraviolet / extreme ultraviolet
$h\nu$ / E_{ph}	photon energy
**eKE / KE**	(electron) kinetic energy
$eKE_{(meas)}$	as-measured electron kinetic energy
**VIE**	vertical ionization energy
**(V)BE**	(vertical) binding energy
VIE_{vac}	VIE with respect to the vacuum energy level
VIE_{EF}	VIE with respect to the Fermi level
$VIE_{EF,1b1}$	VIE of neat water's $1b_{1peak}$ with respect to the Fermi level
$VIE_{EF,solute}$	VIE of the [solute] peak with respect to the Fermi level
$VIE_{vac,1b1}$	VIE of neat water's $1b_{1peak}$ with respect to the vacuum level
$VIE_{vac,solute}$	VIE of the [solute] peak with respect to the vacuum level
$VIE_{vac,O1s}$	VIE of the O 1s core-level for neat water with respect to the vacuum level
$E_{cut(s)}$	real cutoff energy of the sample spectrum
$E_{cut(A)}$	setup / analyzer-dependent cutoff energy
$\Delta E_{g\text{-}l}$	energy distance between gas and liquid peaks
$\Delta E_{l\text{-}l}$	energy distance between liquid solute and solvent peaks
ΔE_{w}	energetic width of the spectrum = distance between cutoff and peak features in neat water
$\Delta E_{w(sol)}$	energetic width of the spectrum for an aqueous solution
E_{v}^{∞}	theoretical vacuum energy level at infinity, far away from any matter
E_{v}^{loc}	local vacuum energy level above the liquid's surface
E_{v}^{det}	local vacuum energy level of the detector / experimental setup
E_{F}	Fermi energy or Fermi level
$e\Phi_{water}$	work function of neat liquid water
$e\Phi_{sol}$	work function of an aqueous solution
$e\Phi_{A}$	work function of the analyzer / experimental setup
$\Delta e\Phi$	work function difference / contact potential between sample and experimental setup
Φ_{str}	streaming potential of a flowing liquid
I_{str}	streaming current of a flowing liquid
μ	chemical potential; equal to E_F
$\bar{\mu}$	electrochemical potential
$\phi_{s,jet}$	total extrinsic surface potential of the liquid jet
χ^{d} / $e\varphi_{outer}$	intrinsic interfacial dipole potential / outer (Volta) potential
$s_{distort}$	bias-voltage-induced energy-distortion scaling factor

1 Useful Considerations on the Presentation of Electron Binding Energies in PES

We re-evaluate what should be the most useful energy-axis presentation used in condensed-phase PE spectroscopy, as recently discussed in the context of core-level studies.[1] This appears to be timely, partially triggered by specific phenomena encountered in aqueous-solution measurements. If we consider PE spectroscopy as a reflection of the photoelectric effect,[2] implying the detection of a direct photoelectron, the commonly applied VIE (or equivalently binding energy, BE) axis (with reference to E_v or E_F, depending on context) has been advised, dating back to the early days of PE spectroscopy and practiced in text books.[3] Here, one makes use of the (simplified) relationship: VIE = KE – hν. Although convenient, this common practice is correct only if the spectra have no dependence on photon energy. When final-state effects (such as photoionized-state vibrational progressions in molecular spectra) are considered, a direct correspondence between KE and VIE breaks down. In molecular spectra, additional axes or markers are often introduced to indicate peak structures originating from the same electronic configuration. In a strict sense, values should thus be reported as KEs, while at the same time the peaks in this progression can be shown on a VIE scale. Similarly, in X-ray PE spectra, the occurrence of additional signals originating from autoionization processes no longer justifies the application of a VIE scale, as such features most often have a fixed KE (which is measured in the experiment) due to their very nature and become photon-energy-dependent when displayed on a VIE scale.[4]

Arguably, the applicability of a VIE scale is seriously questionable when presenting PE spectra from condensed-phase samples. Here, there are a plethora of final-state effects, starting from satellite features due to plasmon loss, (charge-transfer) multiplets, or screening, as well as peak-skewing mechanisms due to, *e.g.*, electron-hole interactions, and resonantly enhanced features that only appear at certain photon energies.[3] These extra signal contributions are incidentally assigned a "meaningless" VIE value when such a scale is globally adopted, and have to be marked and painstakingly discussed. While such discussions can be accommodated, the benefits of assigning a global VIE scale widely disappear. For instance, incidental assignment of a VIE scale to inelastically scattered electrons is incorrect *per se*. This problem is exacerbated when the low KE tail, LET, signal exhibits peak-like enhancements, as observed for the liquid-water case.[5] We thus recommend a more rigorous way of displaying energy scales of PE spectra, as indicated in Figs. 1, 5 and 6 in the main text. PE spectra should be plotted on an absolute or relative KE scale which may be corrected for experiment-specific effects. Then, a second energy scale is introduced which marks calibrated ionization or binding energies of relevant PE features. Additional scales may be employed to mark vibrational progressions or shifted features, *e.g.*, satellite features at a constant energy offset. Indeed, other data processing steps may be required in advance of the proposed procedure. For example, spectra measured using ToF spectrometers have to be converted from flight time to kinetic energy scales with assumptions or calibrations made for the ionization time zero and the particular geometrical arrangement of the flight tube. Even HEA spectrometers have to be pre-calibrated using known gaseous PE features to measure the correct kinetic energy, which remains an imperfect process, with even small changes in sample placement or other changes to the experimental setup potentially slightly altering the measured eKE. In practice, associated corrections of the eKE scale may be applied to the data during analysis.

2 Extrinsic potentials occurring with liquid jets

Here, we briefly comment on the various sources of extrinsic potentials occurring in experiments with free-flowing liquid jets (LJs). There are three major components: the photoionization-induced surface potential (arising from charge-buildup due to water's insulating properties), the contact potential between the liquid and the detector $\Delta\Phi$, and the streaming potential (which has its origin in kinematic charging of the injected LJ) all of which lead to an electric field between the surface of the jet and the grounded analyzer orifice.[5-7] Often,

extrinsic surface potential and streaming potential terms are used interchangeably in the LJ-PES literature, as no experimental distinction is possible; here we use the extrinsic surface-potential term to describe the potential associated with external parameters, $\phi_{s,jet}$. The magnitude of the three aforementioned surface-potential contributions can be influenced by adding an electrolyte to the solution. Notably, a sufficient amount of electrolyte suppresses net jet charging by increasing the conductivity of the liquid, but an overdosing of electrolyte may lead to non-zero streaming potentials.[7-9] A temperature and flow-rate dependence has been additionally identified.[8, 10] The resulting electric field between the jet and the analyzer will change if $\phi_{s,jet}$ changes, and exactly this dependence has been used to track *changes* of $\phi_{s,jet}$, as inferred from the energy shifts of $VIE_{vac,1b1(g)}$ (or $\Delta E_{g-l} = VIE_{vac,1b1(g)} - VIE_{vac,1b1(l)}$). We note that the resulting effect, *i.e.*, an energy shift of the gas-phase peaks *in the direct vicinity* of the liquid surface, is indistinguishable from shifts caused by differences in eΦ between the LJ and the analyzer. Establishing a *field-free* condition for the gas-phase referencing in Method 1 thus implies a compensation to zero of *all* these effects combined (with possible associated modification of the intrinsic surface-dipole potential). The so far common procedure to achieve this is to tune the amount of electrolyte (usually NaCl or NaI) until a (on average) field-free condition has been reached between the sample and analyzer. This condition was referred to as streaming-potential compensation,[7] but one may argue that instead a *residual* streaming potential is engineered to exactly compensate other effects such as those originating from contact potential / eΦ differences. We explicitly determine different electrolyte concentrations to achieve *field-free conditions* when applying Method 1 (which was 2.5 mM at room temperature and a flow rate of 0.8 ml/min.) or to achieve *streaming-potential-free* conditions when applying Method 3 (30-50 mM).

3 Accurate determination of ionizing photon energies

We consider methods for the accurate determination of ionizing photon energy (E_{ph}), which is an essential step for the proper implementation of energy referencing Method 2. Our ionizing-radiation source considerations span the most commonly adopted sources: discharge lamps, synchrotron radiation beamlines, and laser-based high harmonic generation (HHG) setups. In this regard, discharge lamp photon energies are intrinsically precisely determined by their sharp atomic transitions. In the case of our helium discharge lamp, a simple grating monochromator, placed between the plasma-discharge chamber and the exit capillary, is used to select a single, desired emission line; each emission line has an intrinsic bandwidth of just 1-2 meV around the associated transition energy. This makes these sources ideal for calibration measurements, provided the photon energy is high enough to prevent distortion of PE features by inelastic scattering at low kinetic energies (KEs).[11]

Synchrotron beamlines, on the other hand, select a narrow energy window from a rather broad radiation source, which emits either over a wide ('white' light in case of single bending magnets) or narrower, but continuously tunable (wigglers and undulators) spectral range. Both the radiation source and the beamline work in tandem to output the desired photon energy. A multitude of influences, such as mechanical offsets, thermal expansion, aberrations, changes in mirror illumination, and slit settings need to be precisely controlled and checked to assure the correct photon energy output. For this reason, we recommend to measure the photon energy at the end of the beamline at least on the same day, and ideally directly before and/or after the VIE calibration measurement. For this, a dedicated apparatus incorporated within (and towards the end of) the beamline (*e.g.*, gas-phase ionization cell, calibrated X-ray spectrometer etc.) can be used, but it is often more practical to use the photoelectron spectrometer itself. Here two approaches can be adopted. First, the spectrum of the same PE feature can be recorded with both the first and second harmonic outputs of the beamline. Second, the (eKE-integrated) partial electron yield photoabsorption spectrum can be recorded via a short photon-energy scan across a precisely known gas-phase atomic or molecular transition. Both methods were adopted to photon-energy-

calibrate the soft X-ray VIE results (hν = 124 – 950 eV) reported in Figure 4 and Table 1 in the main body of the text (in specific hν cases, these methods were sequentially implemented to doubly-calibrate the soft X-ray energies and cross-check the consistency of the two photon-energy calibration methods). The first approach exploits the fact that any grating monochromator will direct several diffraction orders (hν, 2hν, 3hν etc.) along the beamline at a specific nominal photon energy setting, generally at successively reduced efficiencies for the higher diffraction orders. Thus, each PE feature will appear at multiple KEs and the measurement of both the first and second order PE signals makes it possible to calculate E_{ph} from the energetic separation of the two signals, $E_{ph} = KE_{2nd} - KE_{1st}$ (provided the spectrometer eKE axis is sufficiently linear). This approach has the drawback that higher-order light is often actively suppressed at modern synchrotron radiation beamlines and both the ionization cross sections and spectrometer transmission quickly diminish at higher KEs, generally leading to very weak or even unmeasurable second-order PE signals. Furthermore, broad or noisy spectral features measured with low-flux, residual higher-harmonic sources have the potential to introduce considerable errors in the determination of energetic distances. Also, using liquid-phase features (*e.g.*, the water O 1s core level or solute peaks) for this calibration purpose requires sufficiently equilibrated and stable conditions between the first and second harmonic measurements, as slight energetic shifts caused by changing surface potentials lead to errors in the determination of E_{ph}. The second approach of scanning the photon energy over a known gas-phase resonant transition feature usually does not suffer from such signal-intensity problems. Here, the partial electron yield PE spectra are generally recorded and are subsequently integrated over the eKE axis to produce a proxy for the true X-ray absorption spectrum. The resonances are identified as PE signal enhancements on the E_{ph} energy axis. In the soft X-ray measurements reported in the main body of the text, the $Ar_{(g)}$ $2p_{3/2} \rightarrow 3d$ transition at 246.927 ± 0.001 eV,[12] $CO_{(g)}$ C 1s → π* (v=0) transition at 287.41 ± 0.005 eV,[13] $N_{2(g)}$ N 1s → π_g* (v=0) transition at 400.868 ± 0.001 eV,[14] $CO_{(g)}$ O 1s → π* transition at 534.21 ± 0.09 eV,[15] and $Ne_{(g)}$ 1s → 3p transition at 867.29 ± 0.01 eV[16] were implemented to calibrate the photon energies. Resonant photon energies of 123.464 ± 0.001 eV (2nd harmonic of the beamline was resonant with the $Ar_{(g)}$ $2p_{3/2} \rightarrow 3d$ transition), 246.927 ± 0.001 eV, 400.868 ± 0.001 eV, and 867.29 ± 0.01 eV were accordingly set for PES measurements and the cumulative resonant absorption data was used to produce overall beamline calibrations that could be used to precisely determine the nominally set 650 eV and 950 eV photon energies in addition. Generally, the resonant absorption method has the drawback of necessitating measurements at (or close to) suitably calibrated atomic or molecular transition energies. Notably, it is not possible to accurately determine the photon energy by measuring a single PE spectrum of a known peak alone, as this would not correct for any intrinsic energy offsets in the measured kinetic energy scale of the spectrometer, which cannot be disentangled from offsets in the photon energy, *i.e.*, $KE_{meas} = (E_{ph} + \Delta KE_{error}) - IE_{ref}$.

For laser-based HHG sources, the photon energy notably sensitively depends on the driving laser and gas-cell parameters at the point of harmonic generation,[17-19] as well as the subsequent monochromatizing beamline parameters. Careful, at least daily, photon energy calibrations as well as source-stabilization measures are accordingly generally required to make full use of Method 2 with such sources. With ToF-based spectrometers – which are most often implemented with photon-number-limited, monochromatized (but still relatively broadband) HHG sources – the laser pointing and laser-liquid-jet crossing position in front of the spectrometer entrance aperture has the further effect of impacting the spectrometer eKE calibration. To enable both accurate hν and eKE calibration, the following procedure is suggested. With hν sufficiently in excess of the water or aqueous solution IEs of interest (to avoid deleterious inelastic-scattering-induced effects, *i.e.*, hν ≥ 30 eV), the spectrometer ionization time-zero can often be precisely determined via the reflection of the ionizing radiation towards the electron detector, due to its additional sensitivity to EUV photons. Conveniently, the Fresnel reflection of the HHG beam from a liquid microjet placed in front of a ToF-electron analyzer is sufficient to

generate a significant secondary electron signal at commonly implemented micro-channel plate detectors. The photon arrival time at the LJ and time-zero for the photoemission experiment can therefore be readily calculated based on the measured reflected photon-pulse arrival time at the detector and known or determinable distance-of-flight from the LJ to the detection plane. By subsequently recording gas-phase photoelectron spectra from gas-phase PES peaks – *e.g.*, He 1s, Ne 2p, Ar 3s/3p, Kr 4s/4p, and/or Xe 5p – with well-known VIEs under field-free conditions, the photon energy and field-free spectrometer calibration factor(s) can be precisely determined prior to the absolute liquid-phase VIE measurement using Method 2. Upon switching to a liquid-phase sample, bias-free spectra can be recorded and utilized to subsequently calibrate spectra recorded from a sufficiently negatively biased (and electrically conductive) LJ. Method 2 can then be applied as described in the main body of the text; allowing the spectrometer to equilibrate before recording the LET spectrum, the spectral features of interest, and then the LET spectrum again (to ensure self-consistency of the measurements). To engender the most reliable energy referencing results, we further recommend to cross-check the photon energy calibration with further gas-phase measurements following the liquid-phase experiments.

4 Challenges in measuring detector-transmission corrected spectra from liquid targets

Obtaining absolute intensities from photoelectron spectra is a notoriously difficult task, due to the many different factors affecting the detected count rate. One of these factors is the detection systems ability to register an electron of a given kinetic energy (KE), in short, the energy-dependent electron transmission. This response can be calibrated under certain restrictions, e.g., by using a well-characterized and precisely prepared solid or gaseous reference target, or by using an electron gun over a limited energy range (here, smaller KEs are usually more prone to variations by stray fields). In a hemispherical electron analyzer (HEA) several spectrometer characteristics can affect the intensity output (see e.g., Ref. [20]): Non-linear counting at the detector surface, secondary-electron generation within the detection system, and transmission characteristics of the lens and hemispherical parts of the analyzer (sometimes the latter part alone is referred to as the 'transmission function'). Usually, the characterization has to be repeated for different settings of the HEA, such as the pass energy, different lens apertures, hemisphere entrance slit size etc.

However, in contrast to the aforementioned transmission curve characterizations, which, once done, just have to be applied to measured spectra thereafter, volatile liquid microjets pose several significant additional challenges. A primary factor is the, yet-to-be-quantitatively-determined electron KE-dependent inelastic scattering within the vapor layer above the probed liquid surface. This may lead to energy-dependent attenuation and an additional secondary-electron signal contribution. Consequently, energy-, sample-morphology-, and instrument-dependent modulations of the electron transmission function are expected when a volatile liquid jet sample is implemented. This effect will notably be most severe when flat liquid microjets with surface dimensions of the order of the HEA entrance aperture are utilized, with correspondingly high evaporated gas loads. Although, a benefit of such sample morphologies is a relatively low-sensitivity of the transmission function of the analyzer to the sample position, at least in the plane orthogonal to the electron-collection axis.

For experiments utilizing narrow-diameter cylindrical microjets with lower gas load (like those utilized in this work), a further challenge arises in that the HEA transmission function will sensitively depend on the 3D position of the liquid microjet sample with respect to the HEA entrance aperture. In this case, electrons originate from only a very small target region (a μm-radius, curved liquid surface) and slight misalignment of the liquid jet with respect to the HEA's center axis leads to electron KE-dependent signal intensity variations. Another challenge is encountered due to the tendency of liquid jets to create electric potentials between the liquid surface and the HEA entrance aperture. This is further complicated by the changing surface potentials that arise as

evaporated species gradually adsorb at the inner surface of the sample chamber and the HEA. As the associated fields will be absent or altered when gas- or solid-phase transmission calibrants are implemented, associated calibrations will generally be inapplicable to the liquid-jet measurements.

Finally, depending on the pumping capacity of the HEA, it may be the case that the electron detector (MCP) is operated at elevated pressure when a liquid microjet is implemented. While this is not the case when the efficiently differentially-pumped HEAs used in this work are implemented, elevated analyzer pressures caused by the gas load associated with the volatile target can affect the amplification efficiency of electron detectors. Collectively, these issues make the calibration of the electron transmission function from a liquid jet target an extremely challenging, and potentially impossible, task.

5 Determination procedure for the position of E_{cut} via a tangent

To determine the exact zero-crossing of the spectrum's LET region, the following procedure is employed, as illustrated in Figure SI-4A. The derivative of the data is computed, which peaks at the inflection point of the rising slope of the cutoff. In some cases, a double-structure may arise in the cutoff spectrum, which can originate from variations in the relative alignment of the ionizing source, LJ, and analyzer; here, we always use the lowest-energy peak in the derivative, which gives a consistent E_{cut} value (see Figure SI-4B). If the data is particularly noisy, it can become difficult to reliably extract E_{cut}, since noise spikes may obscure the real derivative peak structure and can lead to small additional errors when determining the tangent anchor point. However, slight smoothing of the derivative (as opposed to the raw data) has proven to give a consistent result if the signal-to-noise ratio remains at an acceptable level. This is exemplified in Figure SI-4C, where the derivative has been slightly smoothed to identify the correct maximum. The derivative curve's first peak center accordingly gives the tangent anchor and its height gives the tangent slope. Finally, the tangent's intercept with the data's *baseline*, which is taken as the intensity at 2-5 eV below the cutoff, is determined. We refer to this point as the 'zero-crossing' even though the real baseline often has a small positive y-value, *e.g.*, because of residual gas-phase PE signal. This procedure has no free parameters.

One could in principle define an alternative, but non-standard, approach to determine E_{cut}, namely the direct use of the LET curves first inflection point, *i.e.*, the tangent anchor. As noted in the main text, in our high-resolution data, this fixing-point has a 30-60 meV offset from E_{cut} determined via the zero-crossing (this increases to ~150 meV in our lower resolution examples). The use of the tangent anchor may have certain benefits for lower-energy-resolution measurements, where a smeared-out LET has the potential to result in a slanted tangent, potentially yielding too low an E_{cut} value. In such cases, the tangent anchor, being very close to the zero-crossing value in the high-resolution data, has the potential to become a more reliable fixing point, albeit with a small additional error. While we highlight some exploratory resolution-dependent results recorded at a ~125 eV photon energy in Fig SI-4C, the experimental resolving power remains at a factor of 1000, even in the lowest-resolution example. An in-depth study of the behavior of the LET curve and the associated inflection point and tangent results at various and lower resolution settings is warranted, with the goal of exploring the applicability of the aforementioned E_{cut} definitions. However, this is beyond the scope of this work.

We offer some associated words of caution on the alternative methods that have been used to determine cutoff positions and their impact on extracted IEs. While we here adopt a definition of E_{cut} based on the common tangent extrapolation method, some authors may instead report the 'midpoint' of the rise of the cutoff intensity or an alternative point on the LET curve itself, which yields somewhat different IE values. Determining E_{cut} via a fit to the LET curve profile or a traversed intensity level inevitably leads to a dependence of E_{cut} on the shape of the LET curve, which varies with and can even be distorted by the experimental conditions. Adopting an

alternative definition of E_{cut} to the tangent approach implemented here creates a discrepancy between the results, positively offsetting the position of E_{cut} and thus all extracted VIEs, in our case amounting to a several 10 to 100 meV shifts. More specifically, as the experimental energy resolution and sharpness of the LET curves decreases, the results obtained using the different cutoff definitions diverge. As noted above, we are currently unable to ascertain whether the lowest-eKE inflection point in the derivative curve or the associated tangent baseline crossing method yields the correct aqueous-phase energetics when lower-energy-resolution conditions are implemented with Method 2. However, we reiterate that this choice has little consequence when we analyze the highest-energy-resolution measurements reported here. We further note that the E_{cut} positions extracted from either inflection points or tangent zero-crossing points in the LET data have been found to be experimentally robust.

6 Correction of bias-voltage induced shifts of measured photoelectron kinetic energies

We observed that measured electron kinetic-energy values, eKE_{meas}, are distorted for large (≤-30 V) applied bias voltages, at least for the employed high-pressure-tolerant (HiPP) pre-lens HEAs implemented in this work. The distortion manifests itself as a (predominantly linear) increase of eKE_{meas} by a small scaling factor, $s_{distort}$, *i.e.*, if eKE_{meas} is small the effect is negligible but leads to increasingly large errors at larger eKEs. The scaling factor depends on the implemented settings such as the selected pass energy; a systematic study of these technical details has yet to be performed, however. The factor increasingly impacts the accurate determination of VIEs at high photon energies (large spectral widths, ΔE_w), since the cutoff at low eKEs is less affected than the valence features at high eKEs. Conversely, if ΔE_w is small, the eKE_{meas} values for the cutoff and the valence features are similar, and the error is negligible. Two procedures were employed to determine the scaling factor precisely and correct the resulting VIE values, as explained in the following.

The first procedure relies on a comparison of the energetic position of PE peaks at a known photon energy. By measuring the first and second harmonic spectrum of a specific PE feature, the photon energy can be calculated (see section 3) by applying the equation $E_{ph} = KE_{2nd} – KE_{1st}$. Here, KE_{2nd} is usually much larger than KE_{1st}, and if the energy is distorted by an applied bias voltage, then the calculated E_{ph} will be disparate from the real value. This further implies that any photon-energy calibration can be subject to a large error if performed under biased conditions. The same measurement can be repeated in a grounded configuration or E_{ph} can be independently measured, *e.g.*, using gas-phase resonances (again, see section 3), and $s_{distort}$ can then be estimated by comparing the result to the biased case. For example, a gas-phase measurement series defined E_{ph} = 650.033 ± 0.014 eV, while the procedure discussed above yielded 650.216 ± 0.029 eV, which gives $s_{distort}$ = 1.0002842. An exemplary correction calculation is summarized in Table SI-1. This was the highest distortion identified in our measurements, which in the worst case leads to an offset in the VIE value of up to 350 meV at E_{ph} = 950 eV. While the same drawbacks occur as for the high-photon-energy calibration (see section 3), the bias-correction procedure mentioned above is minimally time-consuming and can readily be adopted as an extension of a careful photon-energy calibration procedure.

The second procedure is to measure the desired spectrum with increasing bias-voltage settings and observe the evolution of the resulting VIE value (see Figure SI-6). This yields the voltage onset at which the effect becomes significant and a precise $s_{distort}$ factor can be extracted. If systematically performed for each measurement, a VIE value can be reliably determined free from distortion, either from measurements with a low enough bias voltage or by extrapolation of the behavior. The drawback of this method is that the bias-dependent measurements can be highly time-consuming and the stability of all experimental conditions needs to be precisely maintained during a measurement set. Also, at particularly low bias values, the cutoff may already be

affected by either HEA transmission issues or by the analyzer work function, which obscures the real E_{cut} value and may render an accurate extraction of the VIE at lower bias settings difficult or even impossible.

7 Discussion of the results of previous studies from Olivieri *et al.*[21] and Ramírez[22]

We first discuss the study of Olivieri *et al.*[21], which reported the work function of water from the LET 'midpoint' as $e\Phi_{water}$ = 4.65 ± 0.09 eV somewhat smaller than our value of $e\Phi_{water}$ = 4.73 ± 0.09 eV. The main text already addresses the fact that the 'midpoint' approach to determine E_{cut} will result in higher $e\Phi_{water}$ values (in our high-spectral-resolution cases by several 10 meV up to ~150 meV), further increasing the offset to our result. The methodological inconsistencies and inaccuracies, which we believe lead to the $e\Phi_{water}$ offsets, are summarized below.

The procedure proposed by Olivieri *et al.*[21] requires a precisely known bias potential to determine a 'correction factor' *c* or to relate the measured E_{cut} value to the unbiased onset energy of the spectrum. In principle, an associated error can arise from the output of the voltage supply, with another error arising from the fact that the actual potential at the LJ may differ from the applied bias voltage due to additional resistances in the electrical connection. This becomes apparent in the initial analysis of the O 1s shift in Ref. [21], where the voltage is stated to be just 99% translated into the liquid shift (*e.g.*, a 4.00 V bias leads to a shift of 3.96 eV in the liquid water O1s peak); we observed a similar factor of ~98 % (see Figure SI-3). Apparently, this deviation was not corrected for in the determination of the eΦ difference, and rather the full -40 V bias voltage has been subtracted. This would lead to a rather large error of up to 0.4 eV (40 * 0.99 = 39.6) for all subsequent analyses. On a related note, under typical experimental conditions it can take more than one hour until the equilibration of water (or alternative solution component) adsorption in the spectrometer chamber is established, causing time-dependent changes to the potentials. In the present study, we found that this effect causes energy shifts larger than 100 meV, as shown in Figure SI-2. It is unclear whether or not such behaviors were properly accounted for in the work of Olivieri *et al.* Unfortunately, with their proposed procedure, it is notably impossible to disentangle eΦ differences from additional extrinsic surface potentials, as both will have the same effect, namely a shift and broadening of the gas-phase O1s peak when ionization occurs close to the liquid surface. Such omission bears the immediate risk that the individual effects add up to a rather large error, manifested here as higher O 1s level ionization energies and lower eΦ values, with respect to our determinations: 538.21 ± 0.07 eV versus 538.10 ± 0.05 eV and 4.65 ± 0.09 eV versus 4.73 ± 0.09 eV for $VIE_{vac,O1s}$ and $e\Phi_{water}$, respectively. Furthermore, Olivieri *et al.* claim that the thus far published VIE data on aqueous solutions may be incorrect by values as large as the eΦ difference between the detector and the sample when employing referencing Method 1 (Figure 6). This is clearly not the case for the determination of the lowest ionizing energy of liquid water, associated with the $1b_{1(l)}$ peak position. This VIE should be recorded with sufficiently dilute aqueous solutions, given that the gas-phase referencing method is only (or should only be) applicable after establishing *field-free* conditions, *i.e.*, after compensation of all effects, including any eΦ difference. While similar arguments could be made for highly concentrated aqueous solutions, shifts in the solvent peaks in those cases do not necessarily originate from eΦ differences, but may have their origin in genuine changes of the bulk or interfacial aqueous electronic structure.[23]

Next, we briefly comment on the recent study of Ramírez[22] It was already stated in the main text that their values of $VIE_{EF,1b1(l)}$ = 6.94 eV and eΦ = 4.60 eV reported for a Zobell solution and $VIE_{EF,1b1(l)}$ = 7.06 eV and eΦ = 4.53 eV reported for a 0.1 M KCl solution (associated errors were not reported), are in disagreement with our results for neat liquid water, *i.e.*, with 50 mM NaI dissolved. $VIE_{vac,1b1(l)}$ values of 11.55 eV and 11.59 eV were also respectively reported for the Zobell and KCl solutions, significantly exceeding the 11.33 ± 0.02 eV value reported here. The Ramírez study notably used the LET 'midpoint' (determined by fitting an EMG

function and with its potentially associated flaws, see section 5) to define $e\Phi$ and $VIE_{vac,1b1(l)}$. The corresponding offset of that fixture point with respect to of E_{cut} determined via the tangent method is likely one of the reasons for the offset of Ramírez's[22] values compared to our values. Furthermore, the $1b_1$ peak centroid energy used to determine the distance to E_{cut} was extracted *after* referencing to the Fermi level, which most likely introduced additional offsets to the VIE value, hinging on the correctness of their VIE_{EF} values. Both solutions are unlikely to fully establish streaming-potential free conditions, which would lead to shifts in the $1b_1$-peak to Fermi level distance. For comparison, we would extract a similarly high VIE_{EF} of ~7.0 eV from an aqueous solution with a salt concentration of just a 2.5-5.0 mM, far away from the $I_{str} = 0$ case identified in our work (compare to Figure SI-7). Finally, the same fixed bias voltage of -20.58 V was apparently used to correct all spectra, regardless of solution, despite the fact that some voltage drop inevitably occurs across the liquid (compare Figure SI-3). This voltage drop ultimately depends on the electrical conductivity of the liquid and the distance to the source (which can be assumed to remain constant, however). Using the same fixed value may thus introduce variable errors when applied to spectra of different aqueous solutions.

Figures

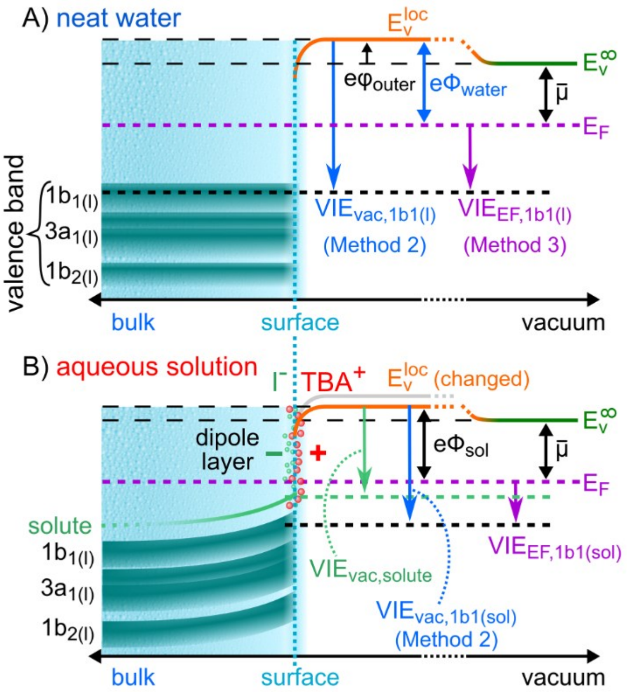

Figure SI-1: Schematic energy diagram highlighting the potentials and reference energy levels of relevance to liquid-phase PES experiments, adapted here from Ref. [24] for a neat liquid-water sample (A) and an aqueous solution of TBAI (B). In panel (B), TBA^+ and I^- ions accumulate, and change the charge distribution, at the surface layer. Adsorbing strong electron donors or acceptors on semiconducting surfaces induces band bending. The Fermi level within the solution stays fixed (which is termed Fermi-level 'pinning') at its bulk value. PE spectroscopy accesses the interface where the bands are bent, leading to the observed large shift and slight broadening of the $TBAI_{(aq)}$ solution bands.

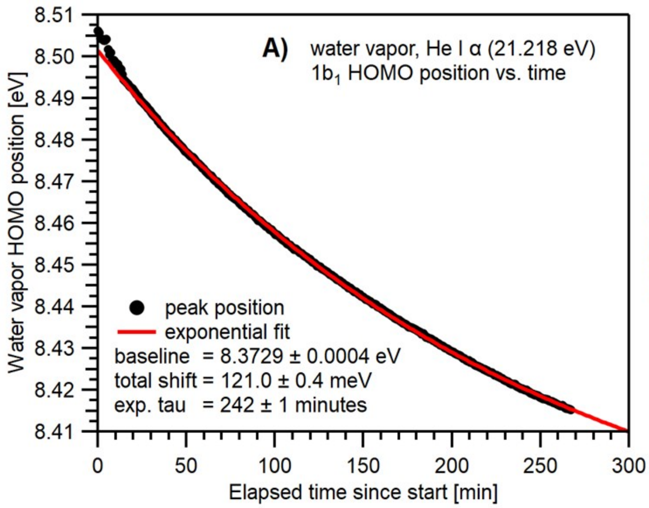

Figure SI-2: Water $1b_{1(g)}$ HOMO ionization peak shift after introducing water vapor via a gas nozzle (~10^{-3} mbar of N_2-equivalent standard pressure at the sensor, *i.e.*, much higher at the nozzle orifice) into a clean experimental setup. **A)** Position of the $1b_{1(g)}$ vibrational-ground-state ionization peak versus elapsed time after introducing the gas. **B)** PE spectra at the start and the end of this measurement series. The observed shift in this particular example is about 120 meV, but will depend on the initial conditions of the chamber and the absolute gas pressure in the vicinity of the detector entrance. The shift evolves over several hours after introduction of the water vapor; an exact energy reference would only be possible after waiting until the system has equilibrated. Accordingly, it is essential that steady-state, equilibrated conditions are established before any extrinsic perturbing potentials are eliminated or compensated – through electrolyte addition or sample biasing – and associated $\Delta E_{g\text{-}l}$ measurements are performed.

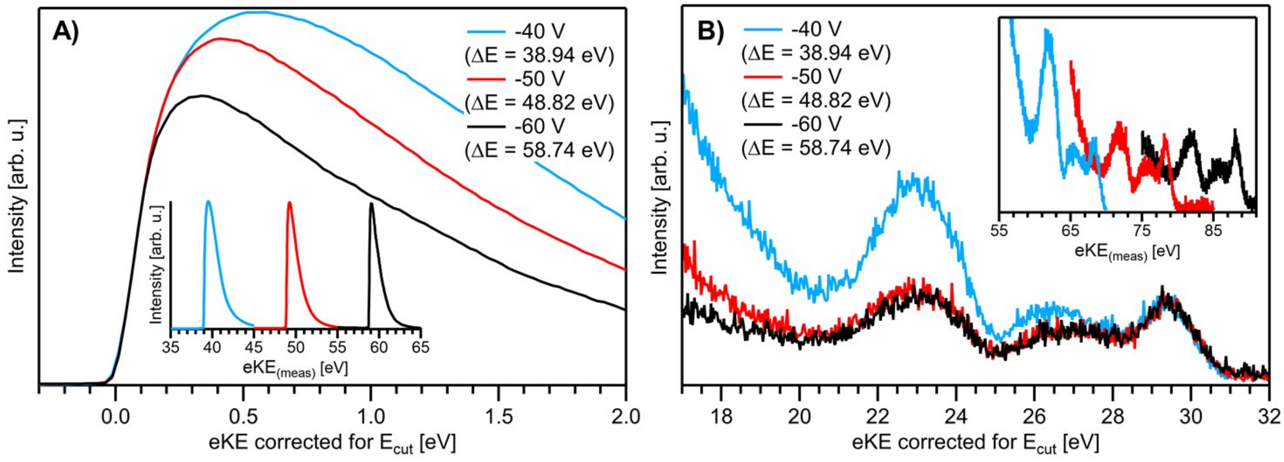

Figure SI-3: Demonstration of the rigid energy shift of all liquid features with increasing bias voltage. The liquid water spectrum is measured with He II α emission (40.814 eV) under the influence of -40 V (blue), -50 V (red), and -60 V (black) bias voltages, as directly applied to the LJ with respect to the grounded PES apparatus. **A)** LET and **B)** valence band regions measured as separate spectra to increase the measurement times and signal-to-noise ratio over the spectral regions of interest. In both panels A) and B) the energy shift was compensated after via the tangent method; the LET curves are shown intensity normalized to yield the same tangent slope for better comparability. The bias voltage leads to an energy shift of all liquid spectral features which is compensated

for at each setting to produce an E_{cut} position at zero on the bottom energy axis (the insets show the uncompensated spectra for comparison); both the LET and valence regions are found to shift by the same amount. Note that the sample must be sufficiently conductive to engender this behavior, which is ensured by dissolving a sufficient amount (~50 mM) of salt in an aqueous sample. The bias voltage is translated ~97.5% into an eKE shift in this case; internal resistances and the voltage accuracies of the power supply cause the reduced value. This is, however, of no relevance for the absolute referencing method, Method 2, where knowledge of the voltage or eKE shift values are not required. We observe a change in the LET curve's shape towards higher eKE's (panel A) under the employed conditions, which however is inconsequential to the shape and inflection point of the cutoff feature. Specifically, the point that determines the cutoff energy (the baseline crossing point of the tangent defined by the spectral derivative; see Figure 1 and SI-4) leads to an almost identical ΔE_W value within our error bars, *i.e.*, our method is invariant to the particular LET shape beyond the initial rise.

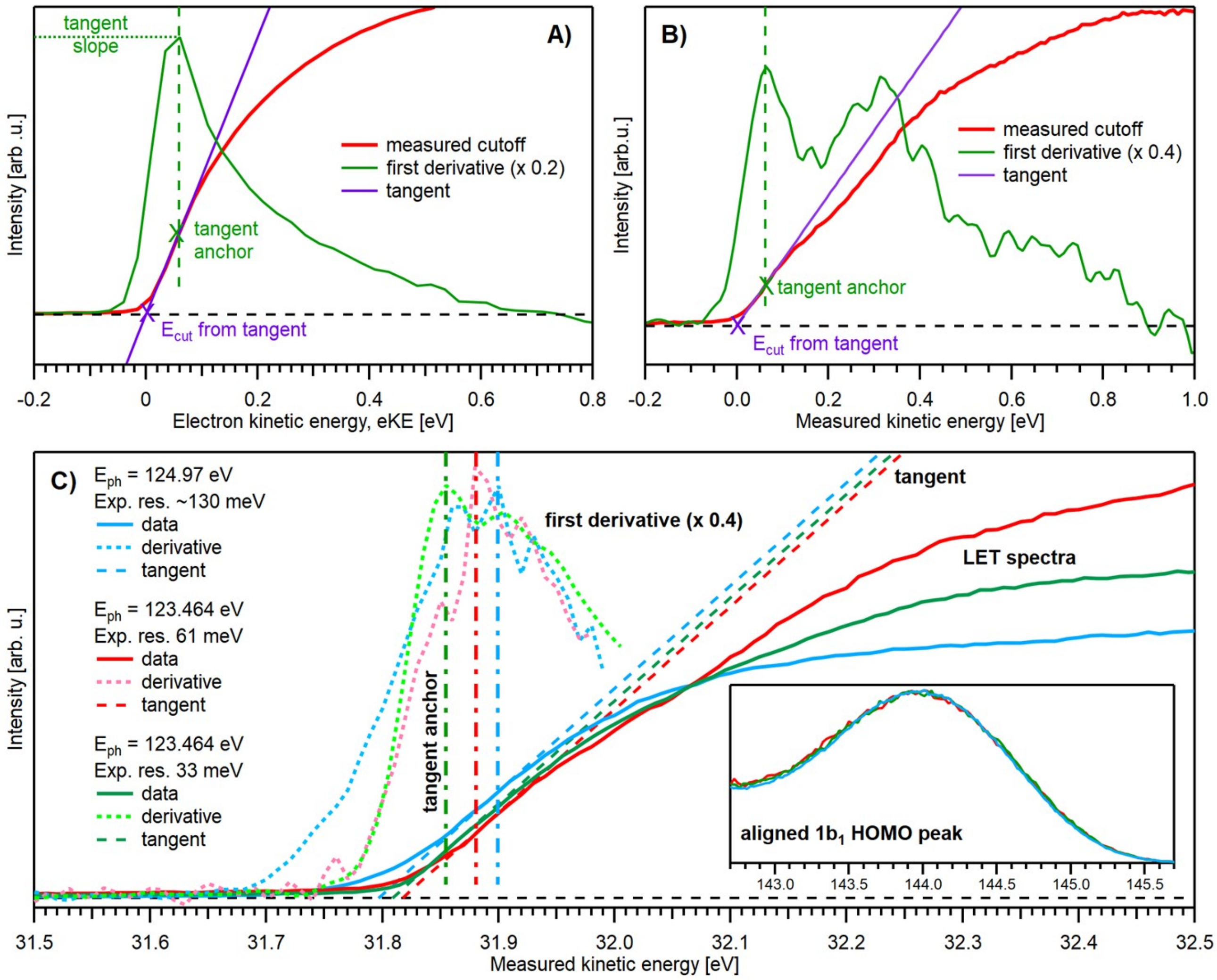

Figure SI-4: Procedure for extracting the LET cutoff position, E_{cut}, via the tangent method exemplified using PE spectra measured with He IIα emission (40.814 eV) in panel A and synchrotron radiation (~123.5-125 eV) in panel B; the bias voltage was -20 V in panel A and -32 V in panels B and C. **A)** The first derivative (green) of the measured data (red) is calculated. The derivative's first maximum (= inflection point with maximum change in slope in the data) automatically determines the tangent anchor point and slope; no free parameters

exist in such a determination. The position of intersection with the baseline (= signal intensity at lower energies below the LET, if non-zero), which determines E_{cut}, is calculated from the tangent equation. E_{cut} defines the energy scale of the photoelectron after leaving the liquid as eKE = 0. **B)** exemplifies data with slight intensity variations in the LET curve, yielding multiple maxima in the derivative. Irrespective of such shape variations, the same procedure as in A), *i.e.*, using the derivative's first maximum, gives the correct E_{cut}. Here, the derivative was additionally smoothed using a 3-point binominal smoothing to reduce noise. **C)** LET spectra, derivative and determined tangents from different beamtimes, spanning 8 months, and with different experimental resolutions of approx. 130 meV (blue), 61 meV (red), and 33 meV (green). Here, the spectra were aligned in a way which maximizes overlap of the $1b_1$ HOMO peak in the associated valence band spectra (see inset), where the different photon energy for the blue spectra was taken into account; this accounts for differences in the applied bias value and makes it possible to directly compare the LET spectra. Furthermore, the spectra were scaled to give an equal slope for all tangents, which makes it easy to see slight shifts in the determined positions. It can be seen that the tangent method is exceptionally robust, with E_{cut} values being stable within 20-30 meV. While the tangent anchor is also very stable within ~50 meV, it is apparent that the tangent zero-crossings and tangent anchor points diverge, with the latter shifting slightly towards higher eKEs, as the LET spectrum is broadened by the decreasing resolution.

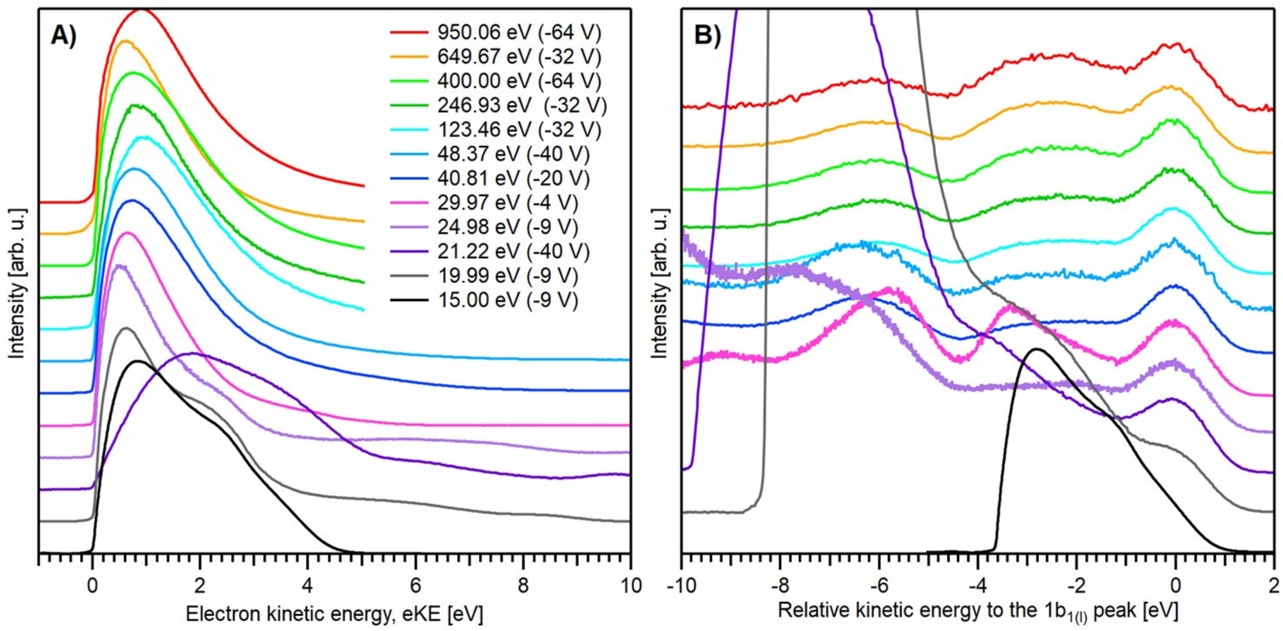

Figure SI-5: Representative PE spectra measured at photon energies spanning 15 eV to 950 eV and analyzed in this study. The small residual gas-phase signal in the cutoff region has been subtracted for the He lamp spectra (21.22 eV, 40.81 eV and 48.37 eV); the gas-phase contribution for all other spectra was negligible. **A)** The LET spectra scaled to yield approximately the same height at the cutoff region, where the spectral cutoff has been aligned to zero eKE. **B)** Spectra measured at the same photon energies as in A) and energetically aligned to the $1b_1$ HOMO ionization peak and vertically scaled to yield approximately the same associated peak height. Valence spectra for the higher photon energies (>48 V) were measured separately from the cutoff spectra under identical experimental conditions and over a limited spectral range (*i.e.,* in different but sequential data acquisitions to the cutoff spectra and making sure to adopt the same HEA pass energy and lens table). This allowed us to maximize acquisition times over the spectral regions of interest and optimize the signal-to-noise ratios.

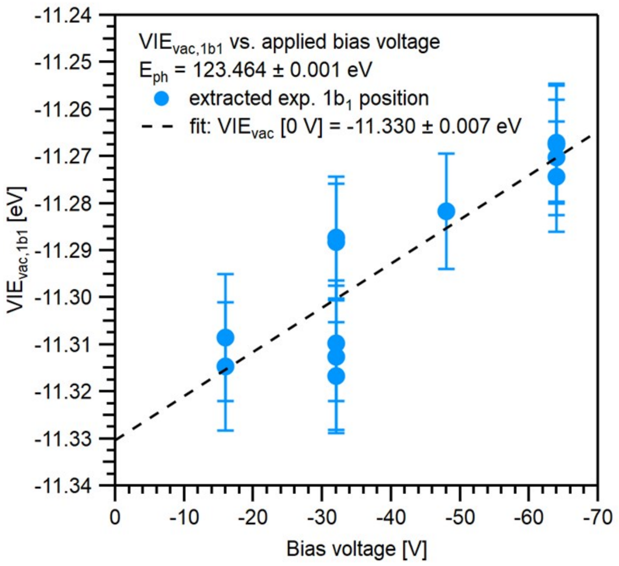

Figure SI-6: Extracted $VIE_{vac,1b1(l)}$ value from a series of measurements over two days with increasing and decreasing applied bias voltage (both directions were probed) as blue dots. The dashed black line is the linear regression of all data points which gives the extrapolated VIE value for 0 V bias voltage. The VIE seemingly decreases with lower bias voltage, which indicates an affected energy scale of the HEA. This deviation gets more severe with higher photon energies, since the energy distance between the cutoff and the valence band features increases, which in turn increases the relative error in the measured eKEs. In cases where only -64 V measurements were performed, the VIE value was corrected by the procedure described in SI section 6.

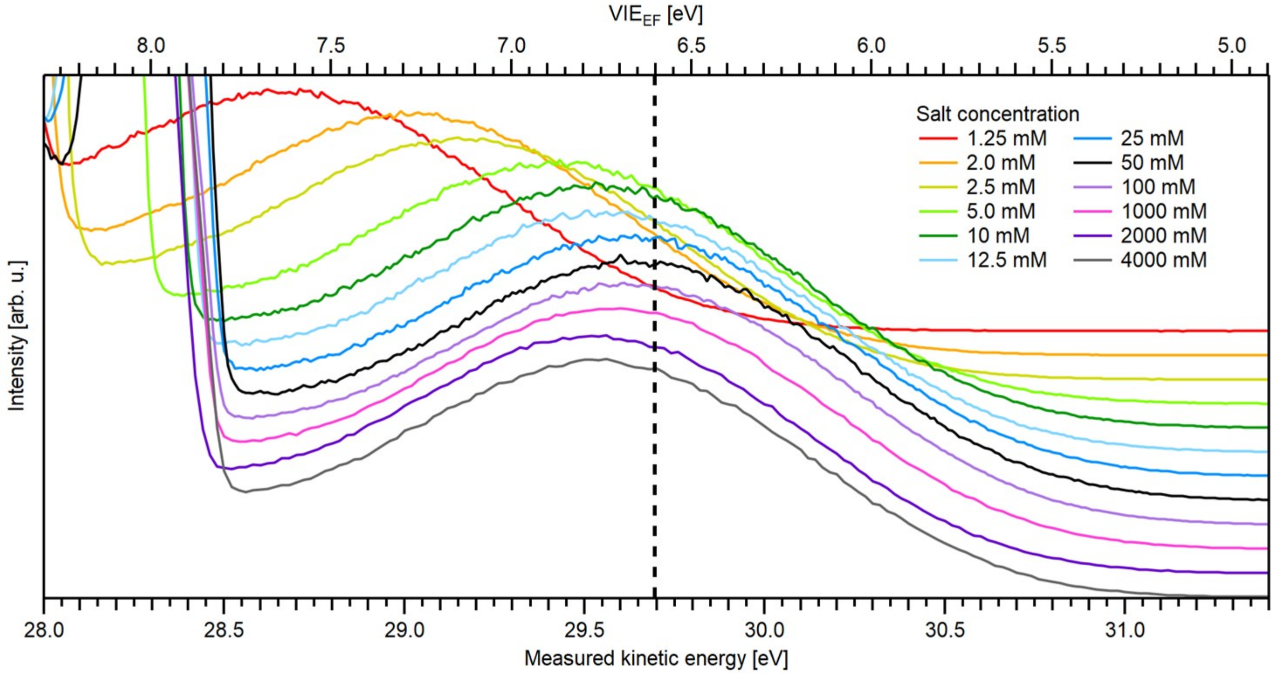

Figure SI-7: Demonstration of the introduced energetic shifts of the gas- and liquid-phase water PE features for different salt concentrations; NaI and NaCl dissolution gives similar results. The liquid spectra were measured with He II α (40.813 eV) radiation and a grounded LJ. A metallic reference PE spectrum defines the top VIE_{EF}

energy scale; the position of the metal reference sample's Fermi edge was unchanged, irrespective of the presence of flowing solutions of different concentrations. It is apparent that the liquid $1b_1$ peak energy shifts widely with respect to the Fermi edge, with a minimum $VIE_{EF,1b1(l)}$ value reached upon dissolution of salt to 25-50 mM concentrations. Higher and lower concentrations shift all liquid PE features towards lower eKE values, which is interpreted as the effect of an uncompensated streaming potential. The optimal concentration of 25-50 mM needed to suppress the streaming current, I_{str}, is in good agreement with previously reported values.[7, 8] At very low concentrations (<1 mM), the sample would be further positively charged due to its insufficient conductivity and means to compensate photoionization-induced electron loss, leading to further retardation of the emitted photoelectrons. Without considering such extrinsic potentials, an arbitrary $VIE_{EF,1b1}$ value would be determined.

Tables

hν (eV)	measured E_{cut} (eV)	measured eKE_{1b1} (eV)	measured $VIE_{vac,1b1(l)}$ (eV)	corrected E_{cut} (eV)	corrected eKE_{1b1} (eV)	corrected $VIE_{vac,1b1(l)}$ (eV)
249.992 ± 0.020	62.368	301.145	-11.23 ± 0.03	62.350	301.059	**-11.28 ± 0.04**
400.007 ± 0.030	62.374	451.182	-11.19 ± 0.03	62.357	451.054	**-11.31 ± 0.04**
650.033 ± 0.030	62.242	701.184	-11.08 ± 0.05	62.224	700.985	**-11.27 ± 0.05**
950.063 ± 0.030	62.252	1001.249	-10.94 ± 0.05	62.234	1000.965	**-11.33 ± 0.08**

Table SI-1: Exemplary comparison of VIE values from a measurement with a bias of -64 V before and after correction with $s_{distort}$ = 1.0002842. The correction factor was applied to both E_{cut} and the valence-band PE spectrum, which then yields the corrected VIE values.

References

1. R. Dupuy, C. Richter, B. Winter, G. Meijer, R. Schlögl and H. Bluhm, *J. Chem. Phys.*, 2021, **154**, 060901.
2. A. Einstein, *Annalen der Physik*, 1905, **17**, 199-206.
3. S. Hüfner, *Photoelectron Spectroscopy: Principles and Applications*, Springer Berlin Heidelberg, 3rd edn., 2010.
4. V. Schmidt, *Reports on Progress in Physics*, 1992, **55**, 1483.
5. B. Winter, R. Weber, W. Widdra, M. Dittmar, M. Faubel and I. V. Hertel, *J. Phys. Chem. A*, 2004, **108**, 2625-2632.
6. M. Faubel, B. Steiner and J. P. Toennies, *J. Chem. Phys.*, 1997, **106**, 9013-9031.
7. N. Kurahashi, S. Karashima, Y. Tang, T. Horio, B. Abulimiti, Y.-I. Suzuki, Y. Ogi, M. Oura and T. Suzuki, *J. Chem. Phys.*, 2014, **140**, 174506.
8. N. Preissler, F. Buchner, T. Schultz and A. Lübcke, *J. Phys. Chem. B*, 2013, **117**, 2422-2428.
9. M. Faubel and B. Steiner, *Ber. Bunsen-Ges. Phys. Chem.*, 1992, **96**, 1167-1172.
10. J. Nishitani, S. Karashima, C. W. West and T. Suzuki, *J. Chem. Phys.*, 2020, **152**, 144503.
11. S. Malerz, F. Trinter, U. Hergenhahn, A. Ghrist, H. Ali, C. Nicolas, C.-M. Saak, C. Richter, S. Hartweg, L. Nahon, C. Lee, C. Goy, D. M. Neumark, G. Meijer, I. Wilkinson, B. Winter and S. Thürmer, *Phys. Chem. Chem. Phys.*, 2021, DOI: 10.1039/d1cp00430a.
12. G. C. King, M. Tronc, F. H. Read and R. C. Bradford, *J. Phys. B: At., Mol. Opt. Phys.*, 1977, **10**, 2479.
13. R. Püttner, I. Dominguez, T. J. Morgan, C. Cisneros, R. F. Fink, E. Rotenberg, T. Warwick, M. Domke, G. Kaindl and A. S. Schlachter, *Phys. Rev. A*, 1999, **59**, 3415.

14. C. T. Chen, Y. Ma and F. Sette, *Phys. Rev. A*, 1989, **40**, 6737(R).
15. R. N. S. Sodhi and C. E. Brion, *J. Electron Spectrosc. Relat. Phenom.*, 1984, **34**, 363-372.
16. A. Müller, D. Bernhardt, A. Borovik Jr, T. Buhr, J. Hellhund, K. Holste, A. L. D. Kilcoyne, S. Klumpp, M. Martins, S. Ricz, J. Veltmann, J. Viefhaus and S. Schippers, *Astrophys. J.*, 2017, **836**, 166.
17. E. Constant, D. Garzella, P. Breger, E. Mével, C. Dorrer, C. Le Blanc, F. Salin and P. Agostini, *Phys. Rev. Lett.*, 1999, **82**, 1668-1671.
18. D. S. Steingrube, T. Vockerodt, E. Schulz, U. Morgner and M. Kovačev, *Phys. Rev. A*, 2009, **80**, 043819.
19. T. Popmintchev, M. C. Chen, A. Bahabad, M. Gerrity, P. Sidorenko, O. Cohen, I. P. Christov, M. M. Murnane and H. C. Kapteyn, *Proc. Natl. Acad. Sci. U. S. A.*, 2009, **106**, 10516-10521.
20. R. C. Wicks and N. J. Ingle, *Rev. Sci. Instrum.*, 2009, **80**, 053108.
21. G. Olivieri, A. Goel, A. Kleibert, D. Cvetko and M. A. Brown, *Phys. Chem. Chem. Phys.*, 2016, **18**, 29506-29515.
22. L. P. Ramírez, *Electronic structure of interfaces studied by in situ real-time XPS*, Doctoral thesis, Sorbonne Université, 2020.
23. M. N. Pohl, E. Muchová, R. Seidel, H. Ali, S. Sršeň, I. Wilkinson, B. Winter and P. Slavíček, *Chem. Sci.*, 2019, **10**, 848-865.
24. W. F. Egelhoff Jr., *Surf. Sci. Rep.*, 1987, **6**, 253-415.
